# Psychiatric and medical comorbidities of eating disorders: findings from a rapid review of the literature

**DOI:** 10.1186/s40337-022-00654-2

**Published:** 2022-09-05

**Authors:** Ashlea Hambleton, Genevieve Pepin, Anvi Le, Danielle Maloney, National Eating Disorder Research Consortium, Phillip Aouad, Sarah Barakat, Robert Boakes, Leah Brennan, Emma Bryant, Susan Byrne, Belinda Caldwell, Shannon Calvert, Bronny Carroll, David Castle, Ian Caterson, Belinda Chelius, Lyn Chiem, Simon Clarke, Janet Conti, Lexi Crouch, Genevieve Dammery, Natasha Dzajkovski, Jasmine Fardouly, Carmen Felicia, John Feneley, Amber-Marie Firriolo, Nasim Foroughi, Mathew Fuller-Tyszkiewicz, Anthea Fursland, Veronica Gonzalez-Arce, Bethanie Gouldthorp, Kelly Griffin, Scott Griffiths, Ashlea Hambleton, Amy Hannigan, Mel Hart, Susan Hart, Phillipa Hay, Ian Hickie, Francis Kay-Lambkin, Ross King, Michael Kohn, Eyza Koreshe, Isabel Krug, Anvi Le, Jake Linardon, Randall Long, Amanda Long, Sloane Madden, Sarah Maguire, Danielle Maloney, Peta Marks, Sian McLean, Thy Meddick, Jane Miskovic-Wheatley, Deborah Mitchison, Richard O’Kearney, Shu Hwa Ong, Roger Paterson, Susan Paxton, Melissa Pehlivan, Genevieve Pepin, Andrea Phillipou, Judith Piccone, Rebecca Pinkus, Bronwyn Raykos, Paul Rhodes, Elizabeth Rieger, Sarah Rodan, Karen Rockett, Janice Russell, Haley Russell, Fiona Salter, Susan Sawyer, Beth Shelton, Urvashnee Singh, Sophie Smith, Evelyn Smith, Karen Spielman, Sarah Squire, Juliette Thomson, Marika Tiggemann, Stephen Touyz, Ranjani Utpala, Lenny Vartanian, Andrew Wallis, Warren Ward, Sarah Wells, Eleanor Wertheim, Simon Wilksch, Michelle Williams, Stephen Touyz, Sarah Maguire

**Affiliations:** 1 InsideOut Institute, Central Clinical School, Faculty of Medicine and Health, Charles Perkins Centre (D17), University of Sydney, Camperdown, NSW 2006 Australia; 2 School of Health and Social Development, Faculty of Health, Deakin University, Geelong, VIC 3220 Australia; 3 Healthcare Management Advisors, Melbourne, VIC Australia; 4 Sydney Local Health District, Camperdown, NSW Australia

**Keywords:** Psychiatric, Medical, Comorbidities, Eating disorders

## Abstract

**Background:**

Eating disorders (EDs) are potentially severe, complex, and life-threatening illnesses. The mortality rate of EDs is significantly elevated compared to other psychiatric conditions, primarily due to medical complications and suicide. The current rapid review aimed to summarise the literature and identify gaps in knowledge relating to any psychiatric and medical comorbidities of eating disorders.

**Methods:**

This paper forms part of a rapid review) series scoping the evidence base for the field of EDs, conducted to inform the Australian National Eating Disorders Research and Translation Strategy 2021–2031, funded and released by the Australian Government. ScienceDirect, PubMed and Ovid/Medline were searched for English-language studies focused on the psychiatric and medical comorbidities of EDs, published between 2009 and 2021. High-level evidence such as meta-analyses, large population studies and Randomised Control Trials were prioritised.

**Results:**

A total of 202 studies were included in this review, with 58% pertaining to psychiatric comorbidities and 42% to medical comorbidities. For EDs in general, the most prevalent psychiatric comorbidities were anxiety (up to 62%), mood (up to 54%) and substance use and post-traumatic stress disorders (similar comorbidity rates up to 27%). The review also noted associations between specific EDs and non-suicidal self-injury, personality disorders, and neurodevelopmental disorders. EDs were complicated by medical comorbidities across the neuroendocrine, skeletal, nutritional, gastrointestinal, dental, and reproductive systems. Medical comorbidities can precede, occur alongside or emerge as a complication of the ED.

**Conclusions:**

This review provides a thorough overview of the comorbid psychiatric and medical conditions co-occurring with EDs. High psychiatric and medical comorbidity rates were observed in people with EDs, with comorbidities contributing to increased ED symptom severity, maintenance of some ED behaviours, and poorer functioning as well as treatment outcomes. Early identification and management of psychiatric and medical comorbidities in people with an ED may improve response to treatment and overall outcomes.

**Supplementary Information:**

The online version contains supplementary material available at 10.1186/s40337-022-00654-2.

## Plain English Summary

The mortality rate of eating disorders is significantly elevated compared to other psychiatric conditions, primarily due to medical complications and suicide. Further, individuals with eating disorders often meet the diagnostic criteria of at least one comorbid psychiatric or medical disorder, that is, the individual simultaneously experiences both an ED and at least one other condition. This has significant consequences for researchers and health care providers – medical and psychiatric comorbidities impact ED symptoms and treatment effectiveness. The current review is part of a larger Rapid Review series conducted to inform the development of Australia’s National Eating Disorders Research and Translation Strategy 2021–2031. A Rapid Review is designed to comprehensively summarise a body of literature in a short timeframe, often to guide policymaking and address urgent health concerns. The Rapid Review synthesises the current evidence base and identifies gaps in eating disorder research and care. This paper gives a critical overview of the scientific literature relating to the psychiatric and medical comorbidities of eating disorders. It covers recent literature regarding psychiatric comorbidities including anxiety disorders, mood disorders, substance use disorders, trauma and personality disorders and neurodevelopmental disorders. Further, the review discusses the impact and associations between EDs and medical comorbidities, some of which precede the eating disorder, occur alongside, or as a consequence of the eating disorder.

**Supplementary Information:**

The online version contains supplementary material available at 10.1186/s40337-022-00654-2.

## Introduction

Eating Disorders (EDs) are often severe, complex, life-threatening illnesses with significant physiological and psychiatric impacts. EDs impact individuals across the entire lifespan, affecting all age groups (although most often they emerge in childhood and adolescence), genders, socioeconomic groups and cultures [1]. EDs have some of the highest mortality rates of all psychiatric illnesses and carry a significant personal, interpersonal, social and economic burdens [2, 3].

Adding to the innate complexity of EDs, it is not uncommon for people living with an ED to experience associated problems such as psychological, social, and functional limitations [2] in addition to psychiatric and medical comorbidities [4–6]. Comorbidity is defined as conditions or illnesses that occur concurrently to the ED. Evidence suggests that between 55 and 95% of people diagnosed with an ED will also experience a comorbid psychiatric disorder in their lifetime [4, 6]. Identifying psychiatric comorbidities is essential because of their potential impact on the severity of ED symptomatology, the individual’s distress and treatment effectiveness [7, 8].

The mortality rate of EDs is significantly higher than the general population, with the highest occurring in Anorexia Nervosa (AN) due to impacts on the cardiovascular system [9] and suicide. [10] Mortality rates are also heightened in Bulimia Nervosa (BN) and Other Specified Feeding and Eating Disorder (OSFED) [11]. Suicide rates are elevated across the ED spectrum, and higher rates are observed in patients with a comorbid psychiatric disorder [10, 12]. Of concern, the proportion of people with an ED not accessing treatment is estimated to be as high as 75% [13], potentially a consequence of comorbidities which impact on motivation, the ability to schedule appointments or require clinical prioritisation (i.e., self-harm or suicidal behaviours) [14]. Further, for many of those diagnosed with an ED who access treatment, recovery is a lengthy process. A longitudinal study found approximately two-thirds of participants with AN or BN had recovered by 22 years follow-up [15]. Although recovery occurred earlier for those with BN, illness duration was lengthy for both groups with quality of life and physical health impacts [15]. Further, less is known regarding the illness trajectory for those who do not receive treatment.

Medical comorbidities associated with EDs can range from mild to severe and life-threatening, with complications observed across all body systems, including the cardiac, metabolic and gastrointestinal, and reproductive systems [5]. These comorbidities and complications can place people at increased risk of medical instability and death [5]. Therefore, understanding how co-occurring medical comorbidities and complications impact EDs is critical to treatment and recovery.

In addition to ED-associated medical comorbidities, EDs often present alongside other psychiatric conditions. Psychiatric comorbidities in people with EDs are associated with higher health system costs, emergency department presentations and admissions [16]. Comorbidities may precede the onset of the ED, be co-occurring, or result from symptoms and behaviours associated with the ED [17, 18]. Individuals with an ED, their carers and care providers often face a complex and important dilemma; the individual with an ED requires treatment for their ED but also for their psychiatric comorbidities, and it can be difficult for treatment providers to determine which is the clinical priority [19]. This is further complicated by the fact that EDs and comorbidities may have a reciprocal relationship, whereby the presence of one impact the pathology, treatment and outcomes of the other.

The current Rapid Review (RR) forms part of a series of reviews commissioned by the Australian Federal Government to inform the Australian National Eating Disorders Research and Translation Strategy 2021–2031 [20]. In response to the impact of psychiatric and medical comorbidities on outcomes, this rapid review summarises the recent literature on the nature and implications of psychiatric and medical comorbidities associated with EDs.

## Methods

The Australian Government Commonwealth Department of Health funded the InsideOut Institute for Eating Disorders (IOI) to develop the Australian Eating Disorders Research and Translation Strategy 2021–2031 [20] under the Psych Services for Hard to Reach Groups initiative (ID 4-8MSSLE). The strategy was developed in partnership with state and national stakeholders including clinicians, service providers, researchers, and experts by lived experience (both consumers and families/carers). Developed through a two-year national consultation and collaboration process, the strategy provides the roadmap to establishing EDs as a national research priority and is the first disorder-specific strategy to be developed in consultation with the National Mental Health Commission. To inform the strategy, IOI commissioned Healthcare Management Advisors (HMA) to conduct a series of RRs to assess all available peer-reviewed literature on all DSM-5 listed EDs.

A RR Protocol [21] was utilised to allow swift synthesis of the evidence in order to guide public policy and decision-making [22]. This approach has been adopted by several leading health organisations including the World Health Organisation [17] and the Canadian Agency for Drugs and Technologies in Health Rapid Response Service [18], to build a strong evidence base in a timely and accelerated manner, without compromising quality. A RR is not designed to be as comprehensive as a systematic review—it is purposive rather than exhaustive and provides actionable evidence to guide health policy [23].

The RR is a narrative synthesis adhering to the PRISMA guidelines [24]. It is divided by topic area and presented as a series of papers. Three research databases were searched: ScienceDirect, PubMed and Ovid/Medline. To establish a broad understanding of the progress made in the field of EDs, and to capture the largest evidence base from the past 12 years (originally 2009–2019, but expanded to include the preceding two years), the eligibility criteria for included studies were kept broad. Therefore, included studies were published between 2009 and 2021, written in English, and conducted within Western healthcare systems or health systems comparable to Australia in terms of structure and resourcing. The initial search and review process was conducted by three reviewers between 5 December 2019 and 16 January 2020. The re-run for the years 2020–2021 was conducted by two reviewers at the end of May 2021.

The RR had a translational research focus with the objective of identifying evidence relevant to developing optimal care pathways. Searches therefore used a Population, Intervention, Comparison, Outcome (PICO) approach to identify literature relating to population impact, prevention and early intervention, treatment, and long-term outcomes. Purposive sampling focused on high-level evidence studies encompassing meta-analyses; systematic reviews; moderately sized randomised controlled studies (RCTs) (n > 50); moderately sized controlled-cohort studies (n > 50); and population studies (n > 500). However, the diagnoses ARFID and UFED necessitated less stringent eligibility criteria due to a paucity of published articles. As these diagnoses are newly captured in the DSM-5 (released in 2013, within the allocated search timeframe), the evidence base is still emerging, and few studies have been conducted. Thus, smaller studies (n =  ≤ 20) and narrative reviews were also considered and included. Grey literature, such as clinical or practice guidelines, protocol papers (without results) and Masters’ theses or dissertations, were excluded. Other sources (which may not be replicable when applying the current methodology) included the personal libraries of authors, yielding two additional studies (see Additional file 1). This extra step was conducted in line with the PRISMA-S: an extension to the PRISMA Statement for Reporting Literature Searches in Systematic Reviews [25].

Full methodological details including eligibility criteria, search strategy and terms and data analysis are published in a separate protocol paper, which included a total of 1320 studies [26] (see Additional file 1: Fig. S1 for PRISMA flow diagram). Data from included studies relating to psychiatric and medical comorbidities of EDs were synthesised and are presented in the current review. No further analyses were conducted.

## Results

The search included articles published in the period January 2009 to May 2021. The RR identified 202 studies for inclusion. Of these, 58% related to psychiatric comorbidities (n = 117) and 42% to medical comorbidities (n = 85). A full list of the studies included in this review and information about population, aims and results can be found in Additional file 2: Tables S3, S4. Results are subdivided into two categories: (1) psychiatric comorbidities and (2) medical complications. Tables 1 and 2 provide high-level summaries of the results.

**Table 1:** Summary of results for psychiatric comorbidities included in the rapid review

Psychiatric comorbidity	Summary
Anxiety disorders (5 studies)	Anxiety disorders are most frequently comorbid condition reported in large population studies [8, 27]. Anxiety disorders often precede onset of EDs [28]. Temperamental factors specifically, anxiety sensitivity and experiential avoidance [29] appear to be associated with anxiety disorder and ED comorbidity
Generalised anxiety disorder (GAD) (2 studies)	Noted evidence of a potential genetic link between EDs and GAD; the presence of one significantly increases the likelihood of the other [8, 30] There were particular links between fasting, excessive exercise, low BMI and comorbid GAD [30]
Social anxiety (4 studies)	Elevated rates of social anxiety across all ED diagnoses; highest in bulimia nervosa (BN), followed by binge eating disorder (BED) and AN-BP (AN—binge—purge subtype). Social anxiety was associated with more severe ED psychopathology and higher body weight [31] and acts as a barrier to accessing ED treatment [32]
Obsessive compulsive disorder (OCD) (5 studies)	The reported comorbidity rates of OCD and EDs were variable [33]. There is symptom overlap between OCD and EDs [34]; with evidence of OCD being related to more severe ED [34]; and treatment of one condition associated with symptom improvement in the other [35]. Further, comorbidity of OCD and EDs appears to be maintained by intrusive thoughts and perfectionism [36]
Major depressive disorder (MDD)/depression (10 studies)	Disordered eating may develop concurrently with depressive symptoms. Changes in frontal brain circuits seen in Depression, are also observed in EDs [37]. There is a strong relationship between MDD and EDs [AN, BN, BED and night eating syndrome (NES)]. However, results were mixed regarding the impact of MDD-ED comorbidity on treatment outcomes
Bipolar disorder (BD) (11 studies)	Notable rates of comorbidity between BD and EDs were reported, however evidence about the frequency of this association was mixed, ranging between 1.9% to as high as 35.8% [38–40]. BD was seen most in EDs which consist of a binge and/or purge symptom profile and comorbidity appears to impact on ED symptom severity, poorer daily and neuropsychological functioning
Personality disorders (PDs) (9 studies)	The association between any type of ED and PDs, was significantly higher than the general population [41]. For specific PDs, the proportions of paranoid, borderline, avoidant, dependant and obsessive–compulsive PD were significantly higher in EDs than the general population. The comorbidity between particular EDs and PDs appeared to be associated with common traits [i.e., impulsiveness of BN with borderline personality disorder (BPD), rigidity of AN with obsessive compulsive personality disorder (OCPD)] Comorbidity was associated with greater distress and poorer outcomes. [41, 42]
Substance use disorders (SUD) (5 studies)	The prevalence rates of substance use in EDs were higher than the general population. Alcohol, caffeine and tobacco were the most frequently reported substances used in ED populations [43]. SUD was more frequently comorbid among individuals with binge/purge type EDs [44]
Psychosis and schizophrenia (3 studies)	A limited area of research; the majority focussed on NES–12% of participants with schizophrenia also met criteria for NES [45]. The actual comorbidity between psychotic disorders and ED remains unclear
Body dysmorphic disorder (BDD) (5 studies)	AN and BDD share similar psychopathology and both have a peak onset period in adolescence, although BDD development typically precedes AN [46]. The prevalence rates of BDD among individuals with AN are variable, however BDD contributes to greater symptom severity in individuals with AN
Attention deficit hyperactivity disorder (ADHD) (9 studies)	A positive association between ADHD and disordered eating, particularly between overeating and ADHD [47]. A twofold increased risk of ADHD in individuals with an ED [48] and studies have noted particularly strong associations between ADHD and BN [49, 50]. Children with ADHD were more like to experience an ED or binge, purge, or engage in restrictive behaviours above clinical threshold [51]
Autism spectrum disorder (ASD) (4 studies)	Prevalence rates of ASD are reported to be as high as 22.9% among individuals with EDs, compared with 2%, observed in the general population [52]. There is a high level of symptom cross-over between EDs and ASD. Symptoms of ASD are frequently exhibited by patients with AN [53, 54]. An assessment of common phenomena between ARFID and ASD in children found a shared symptom profile of eating difficulties, behavioural problems and sensory hypersensitivity beyond what is observed in children without ASD [55]
Post traumatic stress disorder (PTSD) (3 studies)	A broad range of prevalence rates between PTSD and EDs have been reported; between 16.1–22.7% for AN, 32.4–66.2% for BN and 24.02–31.6% for BED [56]. EDs might have a functional role to manage PTSD symptoms and reduce negative affect [57]
Suicidality (22 studies)	Suicide is one of the leading causes of death for individuals with EDs [58]. EDs were a significant risk factor for suicide, and evidence suggested a genetic association between the two [59, 60] The risk for suicide attempts was higher for those with BN compared to other EDs, however, the risk of death by suicide was highest in AN [61, 62]
Non-suicidal self-injury (NSSI) (7 studies)	Up to one-third of patients with EDs report NSSI at some stage in their lifetime, with over one quarter having engaged in NSSI within the previous year [63] Higher levels of impulsivity and emotional reactivity among patients with EDs were associated with concomitant NSSI [64, 65]. There were significant differences in the prevalence of NSSI across ED diagnoses, although patients with binge/purge subtype EDs were more likely to engage in poly-NSSI [66]

**Table 2:** Summary of results for medical comorbidities included in the rapid review

Medical Comorbidity	Summary
Cardiovascular Complications (4 studies)	AN has attracted the most research focus given its increased risk of cardiac failure due to severe malnutrition, dehydration and electrolyte imbalances [67]. Mitral valve prolapse impacted in 25% of patients, sinus bradycardia was the most common arrhythmia, pericardial effusion prevalence rates ranged from 15 to 30% [68]. The risk of cardiac arrest, arrhythmias and heart failure was higher in males with AN than females with AN [69]
Cancer (1 study)	An area of limited research. One study noted a worse prognosis with higher mortality rates for individuals with EDs from melanoma, cancers of genital organs and cancers of unspecified sites. However, there was no statistically significant difference in cancer risk compared to the general population [70]
Gastrointestinal disorders (GI) (14 studies)	More than 90% of AN patients report fullness, early satiety, abdominal distention, pain and nausea [68]. The actual cause of the increased prevalence of GI disorders and their contribution to ED maintenance remain poorly understood
Bone health (16 studies)	The RR found evidence for bone loss/poor bone mineral density (BMD) and EDs, particularly in AN. The negative impacts of bone loss are more pronounced in individuals with early-onset AN when the skeleton is still developing [67] and among those who have very low BMI [71], with comorbidity rates as high as 46.9% [71]. However, lowered BMD was also observed among patients with BN [72]
Refeeding syndrome (RFS) (20 studies)	Identified studies focused on individuals admitted to an inpatient unit for restrictive EDs. Noted variable prevalence rates of RFS [73] ranging from 0 to 62% [74]. Some studies noted that the provision of higher caloric feeds led to faster recoveries and shorter admission duration; with no incidence of RFS [75–77]. However, more research is required to establish best practice in this space including high quality RCTs in inpatient samples, and controlling for feeding methods (i.e. NG vs oral feeding)
Metabolic syndrome (11 studies)	Most research regarding metabolic syndrome has been among patients with BN, BED and NES. Both BN and BED have increased risk for type 2 diabetes [78]. The results suggested importance of increased monitoring and treatment of type 2 diabetes in individuals with EDs, particularly BED and NES
Oral health (7 studies)	Despite ED patients reporting an increased concern for dental issues and engaging in more frequent oral hygiene, their oral health was worse [79]; with increased risk of dental erosion, periodontal disease, missing teeth and oral mucosal lesions [80, 81]
Vitamin deficiencies (6 studies)	The impact of prolonged malnutrition in early-onset EDs can also impair brain development, substantially reducing neurocognitive function in some younger patients even after weight restoration [82]
Cognitive functioning (1 study)	Some cognitive functions affected (attention, decision making, memory) by EDs recover following nutritional restoration [83]
Reproductive health (9 studies)	Infertility and higher rates of poor reproductive health are strongly associated with EDs, including miscarriages, induced abortions, obstetric complications, and poorer birth outcomes [84, 85]. Amenorrhea is a known consequence of AN, oligomenorrhea (irregular periods) was common among individuals with BN and BED [86] Further, the RR found higher rates of Poly Cystic Ovarian Syndrome (PCOS), premenstrual syndrome (PMS) and premenstrual dysphoric disorder (PMDD) among the ED population compared to the general population

*ADHD* attention deficit hyperactivity disorder, *AN* anorexia nervosa, *AN-BP* anorexia nervosa–predominantly binge-purge subtype, *AN-R* anorexia nervosa—predominantly restrictive subtype, *ASD* autism spectrum disorder, *BED* binge eating disorder, *BMI* body mass index, *BDD* body dysmorphic disorder, *BN* bulimia nervosa, *GI* gastrointestinal disorders, *GAD* generalised anxiety disorder, *MDD* major depressive disorder, *NES* night eating syndrome, *NSSI* Non suicidal self injury, *OCD* obsessive compulsive disorder, *PCOS* poly cystic ovarian syndrome, *PMS* premenstrual syndrome, *PMDD* premenstrual dysphoric disorder, *PTSD* post traumatic stress disorder, *RFS* refeeding syndrome, *SUD* substance use disorder

## Psychiatric comorbidities

The study of psychiatric comorbidities can assist with developing models of ED aetiology, conceptualising psychopathology and has relevance for treatment development and outcomes. Given that common psychological factors are observed across psychiatric disorders [87], it is not surprising that there are high prevalence rates of co-occurring psychiatric conditions with EDs. Comorbidity rates of EDs and other psychiatric conditions are elevated further in ethnic/racial minority groups [88]. When looking at the evidence from studies conducted with children and young people, one study of children with ARFID found that 53% of the population had a lifetime comorbid psychiatric disorder [89]. It emerged from the RR that research regarding psychiatric comorbidities generally focussed on the prevalence rates of comorbidities among certain ED subgroups, with some also exploring implications for treatment and ED psychopathology.

### Anxiety disorders

Research indicates that EDs and anxiety disorders frequently co-occur [8, 27]. The high prevalence rates of anxiety disorders in the general population are also observed in people with EDs; with a large population study finding anxiety disorders were the most frequently comorbid conditions reported [8]. In a study of women presenting for ED treatment, 65% also met the criteria for at least one comorbid anxiety disorder [28]. Of note, 69% of those endorsing the comorbidity also reported that the anxiety disorder preceded the onset of the ED [28]. Another study explored anxiety across individuals with an ED categorised by three weight ranges (individuals whose weight is in the ‘healthy weight’ range, individuals in the ‘overweight’ range and individuals in the ‘obese’ range). While anxiety was elevated across all groups, the authors did note that individuals in the overweight group reported significantly higher rates of anxiety than individuals within the healthy weight group [90]. One study that explored temperamental factors provided some insight into factors that may mediate this association; anxiety sensitivity (a predictor of anxiety disorders) was associated with greater ED severity among individuals in a residential ED treatment facility [29]. Further, this association was mediated by a tendency to engage in experiential avoidance—the authors noting that individuals with greater ED symptoms were more likely to avoid distressing experiences [29].

#### Generalised anxiety disorder (GAD)

Studies have noted the potential genetic links between EDs and GAD, noting that the presence of one significantly increases the likelihood of the other [8, 30]. Further, there appears to be a relationship between the severity of ED behaviours and the co-occurrence of GAD, with comorbidity more likely when fasting and excessive exercise are present, as well as a lower BMI [30]. The authors noted the particularly pernicious comorbidity of EDs (specifically AN) and GAD may be amplified by the jointly anxiolytic and weight loss effects of food restriction and excessive exercise [30].

#### Social anxiety

A meta-analysis of 12 studies found higher rates of social anxiety across all ED diagnoses, with patients with BN demonstrating the highest rate of comorbidity at 84.5%, followed by both BED and AN-BP both at 75% [31]. High levels of social anxiety were also associated with more severe ED psychopathology [31] and higher body weight [91]. This particular comorbidity may also impact on access to treatment for the ED; a large follow-up study of adolescents found that self-reported social phobia predicted not seeking treatment for BN symptoms [32]. Interestingly, two studies noted that anxiety symptoms improved following psychological treatments that targeted ED symptoms, possibly due to a shared symptom profile [29, 31].

### Obsessive–compulsive disorder

Similarities between the symptoms of Obsessive–Compulsive Disorder (OCD) and EDs, such as cognitive rigidity, obsessiveness, detail focus, perfectionism and compulsive routines have long been reported in the literature [34]. Given the symptom overlap, a meta-analysis sought to clarify the lifetime and current (that is, a current diagnosis at the time of data collection) comorbidity rates of OCD and EDs, noting the lifetime comorbidity rate was 18% and current comorbidity rate was 15% [33]. However, the authors noted that this prevalence may double over longer periods of observation, with some follow-up data demonstrating comorbidity rates of 33% [33]. Prevalence rates of OCD seemed to be highest among people with AN (lifetime = 19% and current = 14%) compared to other ED subtypes. In addition to the symptom crossover, this RR found evidence of a complex relationship between OCD and EDs, including a potential association between OCD and greater ED severity [34].

Network analysis found that doubts about simple everyday things and repeating things over and over bridged between ED and OCD symptoms. Further, a pathway was observed between restricting and checking compulsions and food rigidity as well as binge eating and hoarding. However, as the data was cross-sectional, directional inferences could not be made [36]. An earlier study explored how changes in OCD symptoms impact ED symptoms among an inpatient sample [35]. As was hypothesised, decreases in OCD symptoms accounted for significant variance in decreases in ED symptoms, and this effect was strongest among ED patients with comorbid OCD. The study also found that irrespective of whether patients had comorbid OCD or not, when ED symptoms improved, so did symptoms of OCD [35]. The authors concluded that perhaps there is a reciprocal relationship between OCD and ED symptoms, whereby symptoms of both conditions interact in a synergistic, bidirectional manner, meaning that improvement in one domain can lead to improvement in another [35]. These findings were somewhat supported in a study by Simpson and colleagues (2013), which found exposure and response prevention (a specialised OCD treatment) resulted in a significant reduction in OCD severity, as was expected, and an improvement in ED symptoms. In their study, individuals with BN showed more improvement than those with AN–nevertheless, BMI still increased among those underweight [92].

### Mood disorders

#### Depression and major depressive disorder (MDD)

This RR also found high levels of comorbidity between major depression and EDs. A longitudinal study of disordered eating behaviours among adolescents found that disordered eating behaviours and depressive symptoms developed concurrently [37]. Among the sample, over half the adolescent sample had a depressive disorder. Prevalence rates were similar for AN (51.5%) and BN (54%) [37]. The study also explored the neurological predictors of comorbid depression in individuals with EDs, noting that lower grey matter volumes in the medial orbitofrontal, dorsomedial, and dorsolateral prefrontal cortices predicted the concurrent development of purging and depressive symptoms [37]. The results suggested that alterations in frontal brain circuits were part of a neural aetiology common to EDs and depression [37].

This RR found much support for a strong relationship between depression and ED symptomatology. In a study of patients with AN, comorbid MDD was associated with a greater AN symptom severity [93], and this relationship between the symptoms of MDD and AN was bidirectional in a study of adolescents undergoing treatment for AN, whereby dietary restraint predicted increased guilt and hostility (symptoms of low mood) and fear predicted further food restriction [94]. Further studies noted the association between BN, BED and NES, with a higher prevalence of depression and more significant depression symptoms [95–97]. However, other studies have failed to find support for this association–for example, a Swedish twin study found no association between NES and other mental health disorders [98].

The impact of the relationship between depression and EDs on treatment outcomes was variable across the studies identified by the RR. One study noted the impact of depression on attrition; patients with BN and comorbid depression attending a university clinic had the highest rates of treatment drop-out [99]. However, in a sample of patients with AN, the comorbidity of depression (or lack of) did not impact treatment outcome and the severity of depression was not associated with changes in ED symptoms [100]. This finding was supported in another study of inpatients with AN; pre-treatment depression level did not predict treatment outcome or BMI [101].

#### Bipolar disorders

Notable comorbidity rates between bipolar disorders (BD) and EDs were reported in the literature reviewed, however evidence about the frequency of this association was mixed. Studies noted comorbidity rates of BD and EDs ranging between 1.9% to as high as 35.8% [38–40]. In order to better understand the nature of comorbidity, a recent systematic review and meta-analysis found BD (including bipolar 1 disorder and bipolar 2 disorder) and ED comorbidity varied across different ED diagnostic groups (BED—12.5%, BN—7.4%, AN—3.8%) [102]. However, the authors noted the scant longitudinal studies available, particularly in paediatric samples. An analysis of comorbidity within a sample of patients with BD identified that 27% of participants also met criteria for an ED; 15% had BN, 12% had BED, and 0.2% had AN [103]. Two other studies noted considerable comorbidity rates of BD; 18.6% for binge eating [104] and 8.8% for NES [105]. Some studies suggested the co-occurrence of BD and EDs were seen most in people with AN-BP, BN and BED—all of which share a binge and/or purge symptom profile [38, 106]. Specifically, BED and BN were the most common co-occurring EDs with BD [40], however, these EDs are also the most prevalent in the population. Therefore, it is unclear if this finding is reflective of the increased prevalence of BN and BED, or if it reflects a shared underlying psychopathology between BD and these EDs [40].

Comorbid ED-BD patients appear to experience increased ED symptom severity, poorer daily and neuropsychological functioning than patients with only a ED or BD diagnosis [107]. In an effort to understand which shared features in ED-BD relate to quality of life, one study assessed an adult sample with BD [108]. Binge eating, restriction, overevaluation of weight and shape, purging and driven exercise were associated with poorer clinical outcomes, quality of life and mood regulation [108]. Additionally, a study of patients undergoing treatment for BD noted patients with a comorbid ED had significantly poorer clinical outcomes and higher scores of depression [109]. Further, quality of life was significantly lower among patients with comorbid ED-BD [109]. The comorbidity of ED and BD has implications for intervention and clinical management, as at least one study observed higher rates of alcohol abuse and suicidality among patients with comorbid ED and BD compared to those with BD only [40].

### Personality disorders

This RR identified limited research regarding the comorbidity between personality disorders (PD) and EDs. A meta-analysis sought to summarise the proportion of comorbid PDs among patients with AN and BN [41]. There was a heightened association between any type of ED and PDs, and this was significantly different to the general population. For specific PDs, the proportions of paranoid, borderline, avoidant, dependant and obsessive–compulsive PD were significantly higher in EDs than in the general population. For both AN and BN, Cluster C PDs (avoidant, dependant and obsessive–compulsive) were most frequent. The authors noted that the specific comorbidity between specific EDs and PDs appears to be associated with common traits—constriction/perfectionism and rigidity is present in both AN and obsessive–compulsive PD (which had a heightened association), as was the case with impulsivity, a characteristic of both BN and borderline PD [41]. This symptom association was also observed in a study of adolescents admitted to an ED inpatient unit whereby a significant interaction between binge-purge EDs (AN-BP and BN), childhood emotional abuse (a risk factor for PD) and borderline personality style was found [110].

This comorbidity may be associated with greater patient distress and have implications for patient outcomes [41, 42]. Data from a nine-year observational study of individuals with BN reported that comorbidity with a PD was strongly associated with elevated mortality risk [111]. In terms of treatment outcomes, an RCT compared the one- and three-year treatment outcomes of four subgroups of women with BN, defined by PD complexity; no comorbid PD (health control), personality difficulties, simple PD and complex PD [112]. At pre-treatment, the complex PD group had greater ED psychopathology than the other three groups. Despite this initial difference, there were no differences in outcomes between groups at one-year and three-year follow up [112]. The authors suggested this result could be due to the targeting of the shared symptoms of BN and PD by the intervention delivered in this study, and that as ED symptoms improve, so do PD symptoms [112]. Suggesting that beyond symptom overlap, perhaps some symptoms attributed to the PD are better explained by the ED. This was consistent with Brietzke and colleagues’ (2011) recommendation that for individuals with ED and a comorbid PD, treatment approaches should target both conditions where possible [113].

### Substance use disorders

Comorbid substance use disorders (SUDs) are also often noted in the literature as an issue that complicates treatment and outcomes of EDs [114]. A meta-analysis reported the lifetime prevalence of EDs and comorbid SUD was 27.9%, [43] with a lifetime prevalence of comorbid illicit drug use of 17.2% for AN and 18.6% for BN [115]. Alcohol, caffeine and tobacco were the most frequently reported comorbidities [43]. Further analysis of SUDs by substance type in a population-based twin sample indicated that the lifetime prevalence of an alcohol use disorder among individuals with AN was 22.4% [115]. For BN, the prevalence rate was slightly higher at 24.0% [115].

The comorbidity of SUD is considered far more common among individuals with binge/purge type EDs, evidenced by a meta-analysis finding higher rates of comorbid SUD among patients with AN-BP and BN than AN-R [44]. This trend was also observed in population data [116]. Further, a multi-site study found that patients with BN had higher rates of comorbid SUD than patients with AN, BED and Eating Disorder Not Otherwise Specific (EDNOS) (utilised DSM-IV criteria) [117]. Behaviourally, there was an association between higher frequencies of binge/purge behaviours with high rates of substance use [117]. The higher risk of substance abuse among patients with binge/purge symptomology was also associated with younger age of binge eating onset [118]. A study explored whether BN and ED subtypes with binge/purge symptoms predicted adverse outcomes and found that adolescent girls with purging disorder were significantly more likely to use drugs or frequently binge drink [119]. This association was again observed in a network analysis of college students, whereby there was an association between binge drinking and increased ED cognitions [120].

### Psychosis and schizophrenia

The RR identified a small body of literature with mixed results regarding the comorbidity of ED and psychosis-spectrum symptoms. A study of patients with schizophrenia found that 12% of participants met full diagnostic criteria for NES, with a further 10% meeting partial criteria [45]. Miotto and colleagues’ (2010) study noted higher rates of paranoid ideation and psychotic symptoms in ED patients than those observed in healthy controls [121]. However, the authors concluded that these symptoms were better explained by the participant's ED diagnosis than a psychotic disorder [121]. At a large population level, an English national survey noted associations between psychotic-like experiences and uncontrolled eating, food dominance and potential EDs [122]. In particular, these associations were stronger in males [122]. However, the true comorbidity between psychotic disorders and ED remains unclear and further research is needed.

### Body dysmorphic disorder

While body image disturbances common to AN, BN and BED are primarily related to weight and shape concerns, individuals with body dysmorphic disorder (BDD) have additional concerns regarding other aspects of their appearance, such as facial features and skin blemishes [46, 123]. AN and BDD share similar psychopathology and both have a peak onset period in adolescence, although BDD development typically precedes AN [46]. The prevalence rates of BDD among individuals with AN are variable. In one clinical sample of female AN patients, 26% met BDD diagnostic criteria [124]. However, much higher rates were observed in another clinical sample of adults with AN, where 62% of patients reported clinically significant 'dysmorphic concern' [125].

As the RR has found with other mental health comorbidities, BDD contributes to greater symptom severity in individuals with AN, making the disorder more difficult to treat. However, some research suggested that improved long-term outcomes from treatments for AN are associated with the integration of strategies that address dysmorphic concerns [124, 126]. However, there remains little research on the similarities, differences and co-occurrence of BDD and AN, and with even less research on the cooccurrence of BDD and other EDs.

### Neurodevelopmental disorders

#### Attention deficit hyperactivity disorder

Several studies noted the comorbidity between Attention Deficit Hyperactivity Disorder (ADHD) and EDs. A systematic review found moderate evidence for a positive association between ADHD and disordered eating, particularly between overeating and ADHD [47]. The impulsivity symptoms of ADHD were particularly associated with BN for all genders, and weaker evidence was found for the association between hyperactivity and restrictive EDs (AN and ARFID) for males, but not females [47]. Another meta-analysis reported a two-fold increased risk of ADHD in individuals with an ED [48] and studies have noted particularly strong associations between ADHD and BN [49, 50]. In a cohort of adults with a diagnosis of an ED, 31.3% had a 'possible' ADHD [127]. Another study considered sex differences; women with ADHD had a significantly higher lifetime prevalence of both AN and BN than women without ADHD [128]. Further, the comorbidity rates for BED were considerably higher among individuals with ADHD for both genders [128].

Further evidence for a significant association between ADHD and EDs was reported in a population study of children [51]. Results revealed that children with ADHD were more like to experience an ED or binge, purge, or restrictive behaviours above clinical threshold [51]. Another study of children with ADHD considered gender differences; boys with ADHD had a greater risk of binge eating than girls [129]. However, the study found no significant difference in AN's prevalence between ADHD and non-ADHD groups. Further, among patients attending an ED specialist clinic, those with comorbid ADHD symptoms had poorer outcomes at one-year follow-up [130].

#### Autism spectrum disorder

There is evidence of heightened prevalence rates of autism spectrum disorder (ASD) among individuals with EDs. A systematic review found an average prevalence of ASD with EDs of 22.9% compared with 2% observed in the general population [52]. With regards to AN, several studies have found symptoms of ASD to be frequently exhibited by patients with AN [53, 54]. An assessment of common phenomena between ARFID and ASD in children found a shared symptom profile of eating difficulties, behavioural problems and sensory hypersensitivity beyond what is observed in typically developing children (the control group) [55]. While research in this area is developing, the findings indicated these comorbidities would likely have implications for the treatment and management of both conditions [55].

### Post traumatic stress disorder

Many individuals with EDs report historical traumatic experiences, and for a proportion of the population, symptoms of post traumatic stress disorder (PTSD). A broad range of prevalence rates between PTSD and EDs have been reported; between 16.1–22.7% for AN, 32.4–66.2% for BN and 24.02–31.6% for BED [56]. A review noted self-criticism, low self-worth, guilt, shame, depression, anxiety, emotion dysregulation, anger and impulsivity were linked to the association between EDs and trauma [57]. It was suggested that for individuals with trauma/PTSD, EDs might have a functional role to manage PTSD symptoms and reduce negative affect [57]. Further, some ED behaviours such as restriction, binge eating, and purging may be used to avoid hyperarousal, in turn maintaining the association between EDs and PTSD [57].

Few studies have explored the impact of comorbid PTSD on ED treatment outcomes. A study of inpatients admitted to a residential ED treatment service investigated whether PTSD diagnosis at admission was associated with symptom changes [56]. Cognitive and behavioural symptoms related to the ED had decreased at discharge, however, they increased again at six-month follow up. In contrast, while PTSD diagnosis was associated with higher baseline ED symptoms, it was not related to symptom change throughout treatment or treatment dropout [56]. Given previous research identified that PTSD and EDs tend to relate to more complex courses of illness, greater rates of drop out and poorer outcomes, a study by Brewerton and colleagues [131], explored the presence of EDs in patients with PTSD admitted to a residential setting. Results showed that patients with PTSD had significantly higher scores of ED psychopathology, as well as depression, anxiety and quality of life. [131]. Further, those with PTSD had a greater tendency for binge-type EDs.

### Suicidality

Suicide is one of the leading causes of death for individuals with EDs [58]. In a longitudinal study of adolescents, almost one quarter had attempted suicide, and 65% reported suicidal ideation within the past 6 months [37]. EDs are a significant risk factor for suicide, with some evidence suggesting a genetic association between suicide risk and EDs [59, 60]. This association was supported in the analysis of Swedish population registry data, which found that individuals with a sibling with an ED had an increased risk of suicide attempts with an odds ratio of 1.4 (relative cohort *n* = 1,680,658) [61]. For suicide attempts, this study found an even higher odds ratio of 5.28 (relative cohort *n* = 2,268,786) for individuals with an ED and 5.39 (relative cohort *n* = 1,919,114) for death by suicide [61]. A comparison of individuals with AN and BN indicated that risk for suicide attempts was higher for those with BN compared to AN [61]. However, the opposite was true for death by suicide; which was higher in AN compared to BN [61]. This result is consistent with the findings of a meta-analysis—the incidence of suicide was higher among patients with AN compared to those with BN or BED [62].

The higher incidence of suicide in adults with AN [132] is potentially explained by the findings from Guillaume and colleagues (2011), which suggested that comparative to BN, AN patients are more likely to have more serious suicide attempts resulting in a higher risk of death [133]. However, death by suicide remains a significant risk for both diagnoses. As an example, Udo and colleagues (2019) study reported that suicide attempts were more common in those with an AN-BP subtype (44.1%) than AN-R (15.7%), or BN (31.4%) [134]. Further, in a large cohort of transgender college students with EDs, rates of past-year suicidal ideation (a significant risk factor for suicide attempts) was 75.2%, and suicide attempts were 74.8%, significantly higher than cisgender students with EDs and transgender students without EDs [135]. The RR found that the risk of suicidal ideation and behaviour was associated with ED diagnosis and the presence of other comorbidities. Among a community-based sample of female college students diagnosed with an ED, 25.6% reported suicidal ideation, and this was positively correlated with depression, anxiety and purging [136]. In support of this evidence, Sagiv and Gvion (2020) proposed a dual pathway model of risk of suicide attempt in individuals with ED, which implicates trait impulsivity and comorbid depression [137]. In two large transdiagnostic ED patient samples, suicidal ideation was associated with different aspects of self-image between ED diagnoses. For example, suicidal ideation was associated with higher levels of self-blame among individuals with BED, while among patients with AN and OSFED, increased suicidal ideation was associated with a lack of self-love [138, 139].

#### Anorexia nervosa

Amongst adults with AN, higher rates of suicide have been reported amongst those with a binge-purge subtype (25%) than restrictive subtype (8.65%) [58, 140]. Further, comorbid depression and prolonged starvation were strongly associated with elevated suicide attempts for both subtypes [58, 140]. In another study, the risk of attempted suicide was associated with depression, but it was moderated by hospital treatment [93]. Further, suicidal ideation was related to depression. A significant 'acquired' suicide risk in individuals with AN has been identified by Selby et al. (2010) through an increased tolerance for pain and discomfort resultant from repeated exposure to painful restricting and purging behaviours [141].

#### Bulimia nervosa

Further research among individuals diagnosed with BN found an increased level of suicide risk [142]. Results from an extensive study of women with BN indicated that the lifetime prevalence of suicide attempts in this cohort was 26.9% [143]. In one study of individuals diagnosed with severe BN, 60% of deaths were attributed to suicide [144]. The mean age at the time of death was 29.6 years, and predictive factors included previous suicide attempts and low BMI. Further, in a sample of children and adolescents aged 7 to 18 years, higher rates of suicidal ideation were associated with BN, self-induced vomiting and a history of trauma [12].

A large population-based study of adolescents and adults explored the frequency and correlates of suicidal ideation and attempts in those who met the criteria for BN [145]. Suicidal ideation was highest in adolescents with BN (53%), followed by BED (34.4%), other non-ED psychopathology (21.3%) or no psychopathology (3.8%). A similar trend was observed for suicide plans and attempts [145]. However, for adults, suicidality was more prevalent in the BN group compared to no psychopathology, but not statistically different to the AN, BED or other psychopathology groups [145].

Consistent with Crow and colleagues’ (2014) results, in a sample of women with BN, depression had the strongest association with lifetime suicide attempts [146]. There were also associations between identity problems, cognitive dysregulation, anxiousness, insecure attachment and lifetime suicide attempts among the sample. Depression was the most pertinent association, suggesting that potential comorbid depression should be a focus of assessment and treatment among individuals with BN due to the elevated suicide risk for this group [146]. Insecure attachment is associated with childhood trauma, and a systematic review found that suicide attempts in women with BN were significantly associated with childhood abuse and familial history of EDs [58].

#### Binge eating disorder

The RR found mixed evidence for the association between suicidal behaviour and BED. A meta-analysis found no suicides for patients with BED [62]. However, evidence from two separate large national surveys found that a significant proportion of individuals who had a suicide attempt also had a diagnosis of BED [134, 147].

### Non-suicidal self injury

Non-suicidal self-injury (NSSI), broadly defined, is the intentional harm inflicted to one’s body without intent to die [148]. Recognising NSSI is often a precursor for suicidal ideation and behaviour [149], together with the already heightened mortality rate for EDs, several studies have examined the association between EDs and NSSI. Up to one-third of patients with EDs report NSSI at some stage in their lifetime, with over one quarter having engaged in NSSI within the previous year [63]. Similarly, a cohort study [148] found elevated rates of historical NSSI amongst patients with DSM-IV EDs; specifically EDNOS (49%), BN (41%) and AN (26%). In a Spanish sample of ED patients, the most prevalent form of NSSI was banging (64.6%) and cutting (56.9%) [63].

Further research has explored the individual factors associated with heightened rates of NSSI. Higher levels of impulsivity among patients with EDs have been associated with concomitant NSSI [64]. This was demonstrated in a longitudinal study of female students, whereby NSSI preceded purging, marking it a potential risk factor for ED onset [65]. In a study of a large clinical sample of patients with EDs and co-occurring NSSI, significantly higher levels of emotional reactivity were observed [150]. The highest levels of emotional reactivity were reported by individuals with a diagnosis of BN, who were also more likely to engage in NSSI than those with AN [150]. In Olatunji and colleagues’ (2015) cohort study, NSSI was used to regulate difficult emotions, much like other ED behaviours. NSSI functioning as a means to manage negative affect associated with EDs was further supported by Muehlenkamp and colleagues’ [66] study exploring the risk factors in inpatients admitted for an ED. The authors found significant differences in the prevalence of NSSI across ED diagnoses, although patients with binge/purge subtype EDs were more likely to engage in poly-NSSI (multiple types of NSSI). Consistent with these findings, a study of patients admitted to an ED inpatient unit found that 45% of patients displayed at least one type of NSSI [151]. The function of NSSI among ED patients was explored in two studies, one noting that avoiding or suppressing negative feelings was the most frequently reported reason for NSSI [151]. The other analysed a series of interviews and self-report questionnaires and found patients with ED and comorbid Borderline Personality Disorder (BPD) engaged in NSSI as a means of emotion regulation [152].

## Medical comorbidities

The impact of EDs on physical health and the consequential medical comorbidities has been a focus of research. Many studies reported medical comorbidities resulting from prolonged malnutrition, as well as excessive exercise, binging and purging behaviours.

### Cardiovascular complications

As discussed above, although suicide is a significant contributor to the mortality rate of EDs, physical and medical complications remain the primary cause of death, particularly in AN, with a high proportion of deaths thought to result from cardiovascular complications [153]. AN has attracted the most research focus given its increased risk of cardiac failure due to severe malnutrition, dehydration and electrolyte imbalances [67].

Cardiovascular complications in AN can be divided by conduction, structural and ischemic diseases. A review found that up to 87% of patients experience cardiovascular compromise shortly following onset of AN [153]. Within conduction disease, bradycardia and QT prolongation occur at a high frequency, largely due to low body weight and resultant decreased venous return to the heart. Whereas, atrioventricular block and ventricular arrhythmia are more rare [153]. Various structural cardiomyopathies are observed in AN, such as low left ventricular mass index (occurs frequently), mitral prolapse and percardial effusion (occurs moderately). Ischemic diseases such as dyslipidemia or acute myocardial infarction are more rare.

Another review identified cardiopulmonary abnormalities that are frequently observed in AN; mitral valve prolapse occurred in 25% of patients, sinus bradycardia was the most common arrhythmia, and pericardial effusion prevalence rates ranged from 15 to 30%. [68] Sudden cardiac death is thought to occur due to increased QT interval dispersion and heart rate variability. [68] A review of an inpatient database in a large retrospective cohort study found that coronary artery disease (CAD) was lower in AN patients than the general population (4.4% and 18.4%, respectively). Consistent with trends in the general population, the risk of cardiac arrest, arrhythmias and heart failure was higher in males with AN than females with AN [69].

### Cancer

Given that individuals with AN have compromised biology, may avoid medical care, and have higher rates of substance use, research has examined cancer incidence and prognosis among individuals with AN. A retrospective study noted higher mortality from melanoma, cancers of genital organs and cancers of unspecified sites among individuals with AN, however, there was no statistically significant difference compared to the general population [70]. No further studies of cancer in EDs were identified.

### Gastrointestinal disorders

The gastrointestinal (GI) system plays a pivotal role in the development, maintenance, and treatment outcomes for EDs, with changes and implications present throughout the GI tract. More than 90% of AN patients report fullness, early satiety, abdominal distention, pain and nausea [68]. Although it is well understood that GI system complaints are complicated and exacerbated by malnutrition, purging and binge eating [154, 155], the actual cause of the increased prevalence of GI disorders and their contribution to ED maintenance remain poorly understood.

To this end, a review aimed to determine the GI symptoms reported in two restrictive disorders (AN and ARFID), as well as the physiologic changes as a result of malnutrition and function of low body weight and the contribution of GI diseases to the disordered eating observed in AN and ARFID [156]. The review found mixed evidence regarding whether GI issues were increased in patients with AN and ARFID. This was partly due to the relatively limited amount of research in this area and mixed results across the literature. The review noted that patients with AN and ARFID reported a higher frequency of symptoms of gastroparesis. Further, there was evidence for a bidirectional relationship between AN and functional gastrointestinal disorders (FGIDs) contributing to ongoing disordered eating. The review found that GI symptoms observed in EDs develop due to (1) poorly treated medical conditions with GI-predominant symptoms, (2) the physiological and anatomical changes that develop due to malnutrition or (3) FGIDs.

There was a high rate of comorbidity (93%) between ED and FGIDs, including oesophageal, bowel and anorectal disorders, in a patient sample with AN, BN and EDNOS [157]. A retrospective study investigating increased rates of oesophageal cancer in individuals with a history of EDs could not conclude that risk was associated with purging over other confounding factors such as alcohol abuse and smoking [158].

Given that gut peptides like ghrelin, cholecystokinin (CCK), peptide tyrosine (PYY) and glucagon-like peptide 1 (GLP-1) are known to influence food intake, attention has focussed on the dysregulation of gut peptide signalling in EDs [159]. A review aimed to discuss how these peptides or the signals triggered by their release are dysregulated in EDs and whether they are normalised following weight restoration or weight loss (in the case of people with higher body weight) [159]. The results were inconsistent, with significant variability in peptide dysregulation observed across EDs [159]. A systematic review and meta-analysis explored whether ghrelin is increased in restrictive AN. The review found that all forms of ghrelin were raised in AN’s acute state during fasting [160]. In addition, the data did not support differences in ghrelin levels between AN subtypes [160]. Another study examined levels of orexigenic ghrelin and anorexigenic peptide YY (PYY) in young females with ARFID, AN and healthy controls (HC) [161]. Results demonstrated that fasting and postprandial ghrelin were lower in ARFID than AN, but there was no difference between ARFID and AN for fasting and postprandial PYY [161].

Oesophageal and gastrointestinal dysfunction have been observed in patients with AN and complicate nutritional and refeeding interventions [155]. Findings from a systematic review indicated that structural changes that occurred in the GI tract of patients with AN impacted their ability to swallow and absorb nutrients [162]. Interestingly, no differences in the severity of gastrointestinal symptoms were observed between AN-R and AN-BP subtypes [155].

A systematic review of thirteen studies aimed to identify the most effective treatment approaches for GI disorders and AN [163]. An improvement in at least one or more GI symptoms was reported in 11 of the 13 studies, with all studies including nutritional rehabilitation, and half also included concurrent psychological treatment [163]. Emerging evidence on ED comorbidity with chronic GI disorders suggested that EDs are often misdiagnosed in children and adolescents due to the crossover of symptoms. Therefore, clinicians treating children and adolescents for GI dysfunction should be aware of potential EDs and conduct appropriate screening [164]. There has been an emerging focus on the role of the gut microbiome in the regulation of core ED symptoms and psychophysiology. Increased attention is being paid to how the macronutrient composition of nutritional rehabilitation should be considered to maximise treatment outcomes. A review found that high fibre consumption in addition to prebiotic and probiotic supplementation helped balance the gut microbiome and maintained the results of refeeding [165].

### Bone health

The RR found evidence for bone loss/poor bone mineral density (BMD) and EDs, particularly in AN. The high rates of bone resorption observed in patients with AN is a consequence of chronic malnutrition leading to osteoporosis (weak and brittle bones), increased fracture risk and scoliosis [166]. The negative impacts of bone loss are more pronounced in individuals with early-onset AN when the skeleton is still developing [67] and among those who have very low BMI [71], with comorbidity rates as high as 46.9% [71]. However, lowered BMD was also observed among patients with BN [72].

A review [167] explored the prevalence and differences in pathophysiology of osteoporosis and fractures in patients with AN-R and AN-BP. AN-R patients had a higher prevalence of osteoporosis, and AN-BP patients had a higher prevalence of osteopenia (loss of BMD) [167]. Further, the authors noted the significant increase in fracture risk that starts at disease onset and lasts throughout AN, with some evidence that risk remains increased beyond remission and recovery [167]. Findings from a longitudinal study of female patients with a history of adolescent AN found long-term bone thinning at five and ten-year follow-up despite these patients achieving weight restoration [168].

Given this, treatment to increase BMD in individuals with AN has been the objective of many pharmacotherapy trials, mainly investigating the efficacy of hormone replacement [169, 170]. Treatments include oestrogen and oral contraceptives [169–172]; bisphosphonates [169, 173]; other hormonal treatment [174–177] and vitamin D [178]. However, the outcomes of these studies were mixed.

### Refeeding syndrome

Nutritional rehabilitation of severely malnourished individuals is central to routine care and medical stabilisation of patients with EDs [179]. Within inpatient treatment settings, reversing severe malnutrition is achieved using oral, or nasogastric tube feeding. However, following a period of starvation, initiating/commencing feeding has been associated with ‘refeeding syndrome’ (RFS), a potentially fatal electrolyte imbalance caused by the body's response to introducing nutritional restoration [180, 181]. The studies identified in the RR focused predominantly on restrictive EDs/on this population group—results regarding RFS risk were mixed [73].

A retrospective cohort study of inpatients diagnosed with AN with a very low BMI implemented a nasogastric feeding routine with vitamin, potassium and phosphate supplementation [182]. All patients achieved a significant increase in body weight. None developed RFS [182], suggesting that even with extreme undernutrition, cautious feeding within a specialised unit can be done safely without RFS. For adults with AN, aminotransferases are often high upon admission, however are normalised following four weeks of enteral feeding [183, 184]. Further, the RR identified several studies demonstrating the provision of a higher caloric diet at intake to adolescents with AN led to faster recoveries and fewer days in the hospital with no observed increased risk for RFS [75–77]. These findings were also noted in a study of adults with AN [179].

However, the prevalence of RFS among inpatients is highly variable, with one systematic review noting rates ranging from 0 to 62% [74]. This variability was largely a reflection of the different definitions of RFS used across the literature [74]. A retrospective review of medical records of patients with AN admitted to Intensive Care Units (ICUs) aimed to evaluate complications, particularly RFS, that occurred during the ICU stay and the impact of these complications on treatment outcomes [185]. Of the 68 patients (62 female), seven developed RFS (10.3%) [185].

Although easily detectable and treatable, hypophosphatemia (a low serum phosphate concentration) may lead to RFS which is the term used to describe severe fluid and electrolyte shifts that can occur when nutrition support is introduced after a period of starvation. Untreated hypophosphatemia may lead to characteristic signs of the RFS such as respiratory failure, heart failure, and seizures [76, 179, 186–188]. A retrospective case–control study of inpatients with severe AN identified [189]. A retrospective study of AN and atypical AN patients undergoing refeeding found that the risk of hypophosphatemia was associated with a higher level of total weight loss and recent weight loss rather than the patient’s weight at admission [190]. The safe and effective use of prophylactic phosphate supplementation during refeeding was supported by the results from Agostino and colleagues’ chart review study [191], where 90% of inpatients received supplementation during admission.

Higher calorie refeeding approaches are considered safe in most cases, however the steps necessitated to monitor health status are costly to health services [192]. The most cost-effective approach would likely involve prophylactic electrolyte supplementation in addition to high calorie refeeding, which would decrease the need for daily laboratory monitoring as well as shortening hospital stays [75, 191, 192]. A systematic review noted that much of the research regarding refeeding, particularly in children and young people, has been limited by small sample sizes, single-site studies and heterogeneous designs [181]. Further, the differing definitions of RFS, recovery, remission and outcomes leading to variable results. While RFS appears safe for many people requiring feeding, the risk and benefits of it are unclear [193] due to the limited research on this topic. Following current clinical practice guidelines on the safe introduction of nutrition is recommended.

### Metabolic syndrome

Metabolic syndrome refers to a group of factors that increase risks for heart disease, diabetes, stroke and other related conditions [194]. Metabolic syndrome is conceptualised as five key criteria; (1) elevated waist circumference, (2) elevated triglyceride levels, (3) reduced HDL-C, (4) elevated blood pressure and (5) elevated fasting glucose. The binge eating behaviours exhibited in BN, BED and NES have been linked to the higher rates of metabolic syndrome observed in these ED patients [78, 195].

An analysis of population data of medical comorbidities with BED noted the strongest associations were with diabetes and circulatory systems, likely indexing components of metabolic syndrome [196]. While type 1 diabetes is considered a risk factor for ED development, both BN and BED have increased risk for type 2 diabetes [78]. A 16-year observation study found that the risk of type 2 diabetes was significantly increased in male patients with BED compared to the community controls [78]. By the end of the observation period, 33% of patients with BED had developed type 2 diabetes compared to 1.7% of the control group. The prevalence of type 2 diabetes among patients with BN was also slightly elevated at 4.4% [78]. Importantly, the authors were not able to control for BMI in this study. In another study, BED was the most prevalent ED in a cohort of type 2 diabetes patients [197]. Conversely, the prevalence of AN among patients with type 2 diabetes is significantly lower, with a review of national data reporting comorbidity rates to be 0.06% [198].

Metabolic dysfunction was observed in a relatively large sample of individuals with NES, including metabolic syndrome and type 2 diabetes, with women reporting slightly higher rates (13%) than men (11%) [199]. In another group of adults with type 2 diabetes, 7% met the diagnostic criteria for NES [200]. These findings suggested a need for increased monitoring and treatment of type 2 diabetes in individuals with EDs, particularly BED and NES. Another study found BED had a significant impact on metabolic abnormalities, including elevated cholesterol and poor glycaemic control [201].

The RR identified one intervention study, which examined an intervention to address medical comorbidities associated with BN and BED [195]. The study compared cognitive behaviour therapy (CBT) to an exercise and nutrition intervention to increase physical fitness, decrease body fat percentage and reduce the risk for metabolic syndrome. While the exercise intervention improved participants' physical fitness and body composition, neither group reduced cardiovascular risk at one-year follow-up [195].

### Oral health

Purging behaviour, particularly self-induced vomiting, has been associated with several oral health and gastrointestinal dysfunctions in patients with EDs. A case–control study of ED patients with binge/purge symptomology found that despite ED patients reporting an increased concern for dental issues and engaging in more frequent brushing, their oral health was poorer than controls. [79] Further, a systematic review and meta-analysis aimed to explore whether EDs increase the risk of tooth erosion [80]. The analysis found that patients with EDs had more risk of dental erosion, especially among those who self-induced vomiting [80]. These findings were also found in a large cohort study, where the increased risk for BN was associated with higher rates of dental erosion but not dental cavities [81].

However, a systematic review of 10 studies suggested that poor oral health may be common among ED patients irrespective of whether self-induced vomiting forms part of their psychopathology [202]. One study reported that AN-R patients had poorer oral health outcomes and tooth decay than BN patients [203]. Two studies identified associations between NES and poor oral health, including higher rates of missing teeth, periodontal disease [204, 205]. Another study of a group of patients with AN, BN and EDNOS, demonstrated the impact of ED behaviours on dental soft tissue, whereby 94% of patients had oral mucosal lesions, and 3% were found to have dental erosion [206].

### Vitamin deficiencies

The prolonged periods of starvation, food restriction (of caloric intake and/or food groups), purging and excessive exercise observed across the ED spectrum have detrimental impacts on micronutrient balances [207]. The impact of prolonged vitamin deficiencies in early-onset EDs can also impair brain development, substantially reducing neurocognitive function in some younger patients even after weight restoration [82]. Common micronutrient deficiencies include calcium, fat soluble vitamins, essential fatty acids selenium, zinc and B vitamins [183]. One included study looked at prevalence rates of cerebral atrophy and neurological conditions, specifically Wernicke's encephalopathy in EDs and found that these neurological conditions were very rare in people with EDs [208].

### Cognitive functioning

The literature included in RR regarding the cognitive changes in ED patients with AN following weight gain was sparse. It appears that some cognitive functions affected by EDs recover following nutritional restoration, whereas others persist. Cognitive functions, such as flexibility, central coherence, decision making, attention, processing speed and memory, are hypothesised to be impacted by, and influence the maintenance of EDs. A systematic review explored whether cognitive functions improved in AN following weight gain [83]. Weight gain appeared to be associated with improved processing speed in children and adolescents. However, no improvement was observed in cognitive flexibility following weight gain. Further, the results for adults were inconclusive [83].

### Reproductive health

Infertility and higher rates of poor reproductive health are strongly associated with EDs, including miscarriages, induced abortions, obstetric complications, and poorer birth outcomes [84, 85]. Although amenorrhea is a known consequence of AN, oligomenorrhea (irregular periods) was common among individuals with BN and BED [86]. A twin study found women diagnosed with BN and BED were also more likely to have poly cystic ovarian syndrome (PCOS), leading to menstrual irregularities [209]. The prevalence of lifetime amenorrhea in this sample was 10.4%, and lifetime oligomenorrhea was 33.7%. An epidemiological study explored the association of premenstrual syndrome (PMS) and premenstrual dysphoric disorder (PMDD) in women with BN and BED and found prevalence rates as high as 42.4% for PMS and 4.2% for PMDD [210].

Given the increased rates of menstrual irregularities and issues, questions have been raised regarding whether this complication is reversed or improves with recovery. A review of five studies monitoring reproductive functions during recovery over a 6- to 18-year follow up period [211] noted no significant difference between the pooled odds of childbirth rates between the AN and general population—demonstrating that if patients undergo treatment for AN, achieve weight restoration, and continue to maintain wellness, reproductive functions can renormalise [211].

An observational study of women with AN, BN or EDNOS found higher rates of low birth rate, pre-term deliveries, caesarean deliveries, and intrauterine growth restrictions [84]. Increased caesarean delivery was also observed in a large cohort of women diagnosed with BED [212]. However, these women had higher birth weight babies [212]. Further, women with comorbid ED and epilepsy were found to have an increased risk of pregnancy-related comorbidities, including preeclampsia (gestational hypertension and signs of damage to the liver and kidneys*)*, gestational diabetes and perinatal depression [213].

## Discussion

The results from this review identified that the symptomology and outcomes of EDs are impacted by both psychiatric and medical factors. Further, EDs have a mortality rate substantially higher than the general population, with a significant proportion of those who die from an ED dying by suicide or as a result of severe medical complications.

This RR noted high rates of psychiatric and medical comorbidities in people with EDs, with comorbidities contributing to increased ED symptom severity, maintenance of some ED behaviours, compromised functioning, and adverse treatment outcomes. Evidence suggested that early identification and management of psychiatric and medical comorbidities in people with an ED may improve response to treatment and outcomes [29, 35, 83].

EDs and other psychiatric conditions often shared symptoms and high levels of psychopathology crossover were noted. The most prevalent psychiatric comorbidities were anxiety disorders, mood disorders and substance use disorders [8, 100, 119]. perhaps unsurprising given the prevalence of these illnesses in the general population. Of concern is the elevated suicide rate noted across the ED spectrum, the highest observed in AN [58, 140, 149]. For people with AN, suicide attempts were mostly associated with comorbid mood and anxiety disorders [136]. The review noted elevated rates of NSSI were particularly associated with binge/purge subtype EDs [150], impulsivity and emotional dysregulation (again, an example of psychopathological overlap).

With regards to PDs, studies were limited to EDs with binge-purge symptomology. Of those included, the presence of a comorbid personality disorder and ED was associated with childhood trauma [110] and elevated mortality risk [111]. There appeared to be a link between the clinical characteristics of the ED (e.g., impulsivity, rigidity) and the comorbid PD (cluster B PDs were more associated with BN/BED and cluster C PDs were more associated with AN). There was mixed (albeit limited) evidence regarding the comorbidity between EDs and psychosis and schizophrenia, with some studies noting an association between EDs and psychotic experiences [45]. Specifically, there was an association between psychotic experiences and uncontrolled eating and food dominance, which were stronger in males [122]. In addition, the review noted the association between EDs and neurodevelopmental disorders-specifically ADHD—was associated with features of BN and ASD was more prevalent among individuals with AN [53, 54] and ARFID [55].

EDs are complicated by medical comorbidities across the neuroendocrine, skeletal, nutritional, gastrointestinal, dental, and reproductive systems that can occur alongside, or result from the ED. The RR noted mixed evidence regarding the effectiveness and safety of enteral feeding [180, 181], with some studies noting that RFS could be safely managed with supplementation [191]. Research also described the impacts of restrictive EDs on BMD and binge eating behaviour on metabolic disorders [78, 195]. Purging behaviours, particularly self-induced vomiting [79], were found to increase the risk of tooth erosion [81] and damage to soft tissue within the gastrointestinal tract [206]. Further, EDs were associated with a range of reproductive health issues in women, including infertility and birth complications [84].

Whilst the RR achieved its aim of synthesising a broad scope of literature, the absence of particular ED diagnoses and other key research gaps are worth noting. A large portion of the studies identified focused on AN, for both psychiatric and medical comorbidities. This reflects the stark lack of research exploring the comorbidities for ARFID, NES, and OSFED compared to that seen with AN, BN and BED. There were no studies identified exploring the psychiatric and medical comorbidities of Pica. These gaps could in part be due to the timeline utilised in the RR search strategy, which included the transition from DSM-IV to DSM-5. The update in the DSM had significant implications for psychiatric diagnosis, with the addition of new disorders (such as Autism Spectrum Disorder and various Depressive Disorders), reorganisation (for example, moving OCD and PTSD out of anxiety disorders and into newly defined chapters) and changes in diagnostic criteria (including for AN and BN, and establishing BED as a discrete disorder). Although current understanding suggests EDs are more prevalent in females, research is increasingly demonstrating that males are not immune to ED symptoms, and the RR highlighted the disproportionate lack of male subjects included in recent ED research, particularly in the domain of psychiatric and medical comorbidities.

As the RR was broad in scope and policy-driven in intent, limitations as a result of this methodology ought to be considered. The RR only considered ‘Western’ studies, leading to the potential of important pieces of work not being included in the synthesis. In the interest of achieving a rapid synthesis, grey literature, qualitative and theoretical works, case studies or implementation research were not included, risking a loss of nuance in developing fields, such as the association and prevalence of complex/developmental trauma with EDs (most research on this comorbidity focuses on PTSD, not complex or developmental trauma) or body image dissatisfaction among different gender groups. No studies regarding the association between dissociative disorders and EDs were included in the review. However, dissociation can co-occur with EDs, particularly AN-BP and among those with a trauma history [214]. Future studies would benefit from exploring this association further, particularly as trauma becomes more recognised as a risk factor for ED development.

The review was not designed to be an exhaustive summary of all medical comorbidities. Thus, some areas of medical comorbidity may not be included, or there may be variability in the level of detail included (such as, limited studies regarding the association between cancer and EDs). Studies that explored the association between other autoimmune disorders (such as Type 1 Diabetes, Crohn’s disease, Addison’s disease, ulcerative colitis, and coeliac disease) and EDs [215, 216] were not included. Future reviews and research should examine the associations between autoimmune disorders and the subsequent increased risk of EDs, and likewise, the association between EDs and the subsequent risk of autoimmune disorders.

An important challenge for future research is to explore the impact of comorbidity on ED identification, development and treatment processes and outcomes. Insights could be gained from exploring shared psychiatric symptomology (i.e., ARFID and ASD, BN/BED and personality disorders, and food addiction). Particularly in disorders where the psychiatric comorbidity appears to precede the ED diagnosis (as may be the case in anxiety disorders [28]) and the unique physiological complications of these EDs (e.g., the impact of ARFID on childhood development and growth). Further, treatment outcomes would benefit from future research exploring the nature of the proposed reciprocal nature between EDs and comorbidities, particularly in those instances where there is significant shared psychopathology, or the presence of ED symptoms appears to exacerbate the symptoms of the other condition—and vice versa.

The majority of research regarding the newly introduced EDs has focused on understanding their aetiology, psychopathology, and what treatments demonstrate efficacy. Further, some areas included in the review had limited included studies, for example cancer and EDs. Thus, in addition to the already discussed need for further review regarding the association between EDs and autoimmune disorders, future research should explore the nature and prevalence of comorbidity between cancers and EDs. There was variability regarding the balance of child/adolescent and adult studies across the various comorbidities. Some comorbidities are heavily researched in child and adolescent populations (such as refeeding syndrome) and others there is stark child and adolescent inclusion, with included studies only looking at adult samples. Future studies should also address specific comorbidities as they apply to groups underrepresented in current research. This includes but is not limited to gender, sexual and racial minorities, whereby prevalence rates of psychiatric comorbidities are elevated. [88] In addition, future research would benefit from considering the nature of psychiatric and medical comorbidity for subthreshold and subclinical EDs, particularly as it pertains to an opportunity to identify EDs early within certain comorbidities where ED risk is heightened.

## Conclusions

This review has identified the psychiatric and medical comorbidities of EDs, for which there is a substantial level of literature, as well as other areas requiring further investigation. EDs are associated with a myriad of psychiatric and medical comorbidities which have significant impacts on the symptomology and outcomes of an already difficult to treat, and burdensome illness.

## Supplementary Information

**Additional file 1.** PRISMA diagram.

**Additional file 2.** Studies included in the Rapid Review.

## Data Availability

Not applicable—all citations provided.

## Abbreviations

ADHD: Attention deficit hyperactivity disorder
AN: Anorexia nervosa
AN-R: Anorexia nervosa—restricting type
AN-BP: Anorexia nervosa—binge-purge type
ARFID: Avoidant restrictive food intake disorder
ASD: Autism spectrum disorder
BED: Binge eating disorder
BMI: Body mass index
BN: Bulimia nervosa
BPD: Borderline personality disorder
DSM-5: Diagnostic and statistical manual of mental disorders, 5th edition
ED: Eating disorder
GAD: Generalised anxiety disorder
ICD-11: International classification of diseases, 11th edition
MDD: Major depressive disorder
NES: Night eating syndrome
OSFED: Other specified feeding or eating disorder
PTSD: Post-traumatic stress disorder
RR: Rapid review
