# Supplementary figures and images for: Psychiatric and medical comorbidities of eating disorders: findings from a rapid review of the literature
Source: J Eat Disord. 2022 Sep 5;10:132. doi: 10.1186/s40337-022-00654-2 (PMC9442924; doi:10.1186/s40337-022-00654-2)

**Additional File 1 Figure 1. PRISMA diagram**

**
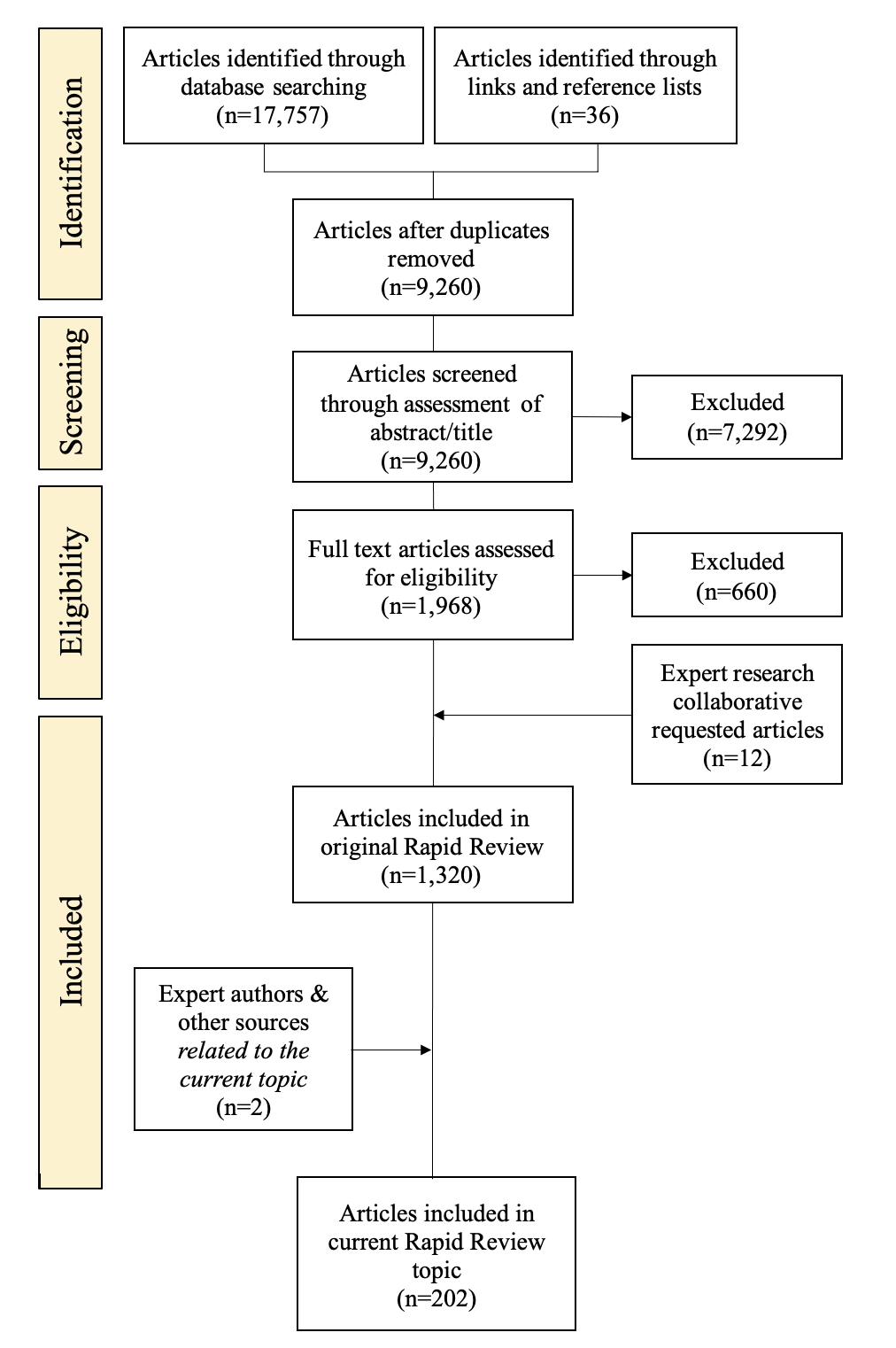
**

Supplement: Supplementary file 1 — Additional file 1. PRISMA diagram. [file 40337_2022_654_MOESM1_ESM.docx]
