# Supplementary material for: Psychiatric and medical comorbidities of eating disorders: findings from a rapid review of the literature
Source: J Eat Disord. 2022 Sep 5;10:132. doi: 10.1186/s40337-022-00654-2 (PMC9442924; doi:10.1186/s40337-022-00654-2)
Supplement: Supplementary file 2 — Additional file 2. Studies included in the Rapid Review. [file 40337_2022_654_MOESM2_ESM.docx]

**Additional File 2** Table 3. Studies included in the Rapid Review.

| **Psychiatric Comorbidities** | | | | | | | | |
| --- | --- | --- | --- | --- | --- | --- | --- | --- |
| **Author** | **Year** | **Title** | **N** | **Country** | **Aim** | **Population** | **Design** | **Outcome** |
| Andersen & Birgegard | 2016 | Diagnosis-specific self-image predicts longitudinal suicidal ideation in adult eating disorders | 1,537 | Sweden | to examine associations between initial self-image and suicidality 12 months after initial contact with a treatment unit (i.e., prediction of suicidality). | Inpatient, Outpatient (Adult, All Genders) | Repeated Measure (without follow-up) | Sociodemographic covariates, self image (SASB introject questionnaire), EDE-Q, mood and anxiety symptoms (CPRS-S-A), Clinical ratings of suicidality |
| Bahji, et al. | 2019 | Prevalence of substance use disorder comorbidity among individuals with eating disorders: A systematic review and meta-analysis | 43 studies | N/A | to comprehensively review the available literature on the comorbidity of substance use disorders (SUD) among individuals with eating disorders (ED). |  | Systematic Review/ Meta-Analysis (combined) | Prevalence rates of substance use disorders |
| Baker, et al. | 2010 | Eating disorder symptomatology and substance use disorders: Prevalence and shared risk in a population based twin sample | 2083 | USA | to examine the prevalence, chronology, and possibility of shared familial risk between SUD and ED symptomatology. | Community (Adult, Women) | Cross-Sectional | Prevalence of substance use disorders in women with AN/BN |
| Balantekin, et al. | 2021 | Overweight and obesity are associated with increased eating disorder correlates and general psychopathology in university women with eating disorders | 690 | USA | to examine how eating disorder (ED) correlates, ED-related clinical impairment, general psychopathology, and ED diagnoses differ across weight statuses in a sample of university women with EDs. | College (Adult, Women) | Cross-Sectional | ED correlates, ED-related clinical impairment, general psychopathology (i.e., depression and anxiety), and ED diagnoses were compared across weight statuses |
| Beilharz, et al. | 2019 | Dysmorphic concern in anorexia nervosa: Implications for recovery | 39 | Australia | to identify the rates of dysmorphic concern in an adult sample diagnosed with AN. | Outpatient (Adult, All Genders, Dx with AN) | Cross-Sectional | Associations between dysmorphic concerns and mental health and ED symptoms |
| Beilharz, et al. | 2017 | A systematic review of visual processing and associated treatments in body dysmorphic disorder | 59 studies | N/A | to summarize our current understanding of visual processing in BDD and review associated treatments. |  | Review (Systematic) | Visual processing abnormalities in BDD, treatment of perception abnormalities |
| Bisset, et al. | 2015 | DSM-5 eating disorder symptoms in adolescents with and without attention-deficit hyperactivity disorder: A population-based study | 2672 | Australia | to examine differences in DSM-5 eating disorder symptoms and partial-syndrome diagnoses at 14-15 years of age in adolescents with and without ADHD in a population-based sample. | Community (Adolescents, All Genders) | Longitudinal (<5yr) | ADHD prevalence and symptoms, Association with ED behaviours |
| Bleck, et al. | 2015 | The Comorbidity of ADHD and Eating Disorders in a Nationally Representative Sample | 12262 | USA | to investigate the association between both clinical and subclinical ADHD and eating disorders via a secondary data analysis of the NLSAH | Community (Young Adult, All Genders) | Cross-Sectional | Association between ADHD and ED symptoms / severity |
| Bodell, et al. | 2013 | Comorbidity-independent Risk for Suicidality Increases with Bulimia Nervosa but not with Anorexia Nervosa | 364 | USA | to examine whether unique associations between eating disorders and suicidality exist and whether potential associations differ by eating disorder diagnosis. | College (Adult, Women) | Longitudinal (>10yr) | Sociodemographic covariates, SCID for Ed dx, lifetime psychiatric diagnoses and lifetime suicidality |
| Boulanger, et al. | 2018 | Binge eating behaviours in bipolar disorders | 145 | France | to investigate the prevalence of BE behaviour and any associations with illness characteristics, anxiety, impulsivity, emotional regulation and eating habits. | Outpatient (Adult, All Genders, Dx with Bipolar Disorder) | Cross-Sectional | Presence of binge eating behaviours in Bipolar Disorder |
| Brewerton & Duncan | 2016 | Associations between Attention Deficit Hyperactivity Disorder and Eating Disorders by Gender: Results from the National Comorbidity Survey Replication | 1686 | USA | to further delineate the differences in the prevalence of lifetime Diagnostic and Statistical Manual of Mental Disorders IV (DSM-IV) ADHD in people with and without current and lifetime DSM-IV ED diagnoses using data from the NCS-R ( | Community (Adult, All Genders) | Cross-Sectional | Associations between ADHD and ED |
| Brewerton, et al. | 2014 | A comparison of women with child‐adolescent versus adult onset binge eating: Results from the National Women's Study | 3006 | USA | to further examine the role of age of onset of binge eating and its correlates. | Community (Adult, All Genders) | Cross-Sectional | Psychological symptoms, dieting, BN and BED symptoms |
| Brewerton, et al. | 2020 | The association of traumatic events and posttraumatic stress disorder with greater eating disorder and comorbid symptom severity in residential eating disorder treatment centres. | 642 | USA | to determine the clinical features indicative of a more severe and complex course have been associated with traumatized ED patients, especially those with PTSD, who may be more likely admitted to residential treatment (RT). | Inpatient (Adult, All Genders) | Cross-Sectional | ED psychopathology, depression, state–trait anxiety, and quality of life. |
| Brietzke, et al | 2011 | Clinical correlates of eating disorder comorbidity in women with bipolar disorder type I | 137 | Brazil | to document current and lifetime ED diagnoses in a well-defined sample of female patients seeking treatment for bipolar disorder type I and determined the association of ED with demographic and clinical features. | Outpatient (Adult, All Genders) | Cross-Sectional | Onset of Bipolar Disorder and symptoms in EDs |
| Brown, et al. | 2018 | The relationship between body mass index, binge eating disorder and suicidality | 14497 | USA | to examine the association between BED, BMI, and suicidality, and assessed whether these relationships varied by gender. | Community (Adult, All Genders) | Cross-Sectional | Sociodemographic covariates, BMI, Suicidality and Suicide Attempts, Binge Eating status from Composite International Diagnostic Interview (CIDI) |
| Buhren, et al. | 2013 | Comorbid Psychiatric Disorders in Female Adolescents with First- Onset Anorexia Nervosa | 148 | Germany | to investigate the rates of comorbid psychiatric disorders, suicidality and self-harm behaviour in adolescent patients with a first onset of AN. | Inpatient (Young People, Women, First onset AN) | RCT | Demographics, weight, height, BMI, duration of illness, SIAB-EX, BDI-II, Kiddie-SADS, EDI-2 |
| Calero-Elvira, et al. | 2009 | Meta-Analysis on Drugs in People with Eating Disorders | 16 studies | N/A | to examine whether drug use (DU) is higher in people with eating disorders (EDs) compared to a healthy control group and to perform a meta-analysis on the literature related to DU in people with EDs. |  | Meta-Analysis | Risk of substance use in EDs |
| Calugi, et al. | 2014 | Depression and treatment outcome in anorexia nervosa | 63 | Italy | to compare the immediate and long-term effect of a cognitive-behaviour therapy program for anorexia nervosa inpatients with and without concomitant Major Depressive Episodes (MDE). | Inpatient (Adult, Women, Dx with AN) | Repeated Measure (with follow-up) | Sociodemographic covariates, ED psychopathology (EDE), general psychopathology (Brief Symptom Inventory) |
| Carriere, et al | 2019 | Relationships between emotional disorders, personality dimensions, and binge eating disorder in French obese adolescents | 115 | France | to explore the association of both emotional disorders and personality dimensions with BED in obese adolescents. | Outpatient (Young People, All Genders) | Cross-Sectional | BED, emotional and personality disorders |
| Cerea, et al. | 2018 | Non-weight related body image concerns and Body Dysmorphic Disorder Prevalence in patients with Anorexia Nervosa | 61 | Italy | to assess the prevalence of BDD and presence of non-weight-related body image concerns in patients with AN. | Inpatient, Outpatient (Adult, Women) | Cross-Sectional | Prevalence of BDD and non-weight-related body image concerns in patients with AN |
| Claes, et al. | 2015 | The Relationship between Non-Suicidal Self-Injury and the UPPS-P Impulsivity Facets in Eating Disorders and Healthy Controls | 338 | Spain | to investigate the prevalence of NSSI in different subtypes of ED patients and healthy controls. | Inpatient (Adult, Women) | Cross-Sectional | Sociodemographic covariates, NSSI, impulsivity, ED Dx (SCID) |
| Claes, et al. | 2010 | The affect-regulation function of non-suicidal self-injury in eating-disordered patients: which affect states are regulated? | 177 | Belgium | (1) to investigate the prevalence of different functions in different types of NSSI in ED patients, (2) to clarify the changes in affective valence and arousal following different types of NSSI, and (3) to examine whether the magnitude of emotional changes associated with NSSI predicts the frequency and chronicity of NSSI. | Inpatient (Adult, Women) | Cross-Sectional | ED Dx, sociodemographics, Self-harm, emotional state before and after self-harm |
| Claes, et al. | 2011 | Male Eating Disorder Patients With and Without Non-suicidal Self-injury: A Comparison of Psychopathological and Personality Features | 130 | Belgium | to investigate the prevalence and comorbidity of non-suicidal self-injury (NSSI) in male eating disorder (ED) patients. | Inpatient (Adult, Men) | Cross-Sectional | ED dx, sociodemographics, self-harm, temperament and character |
| Crow, et al. | 2019 | Factor analysis of the eating disorder diagnostic scale in individuals with bipolar disorder | 1031 | USA | to examine the potential factor structure of the Eating Disorder Diagnostic Scale (EDDS) in a sample of individuals with bipolar disorder. | Attending Bipolar Disorder treatment | Cross-Sectional | Comorbidity between Bipolar Disorder and EDs |
| Crow, et al. | 2014 | Suicidal behaviour in adolescents and adults with bulimia nervosa | 13103 | USA | to examine frequency and correlates of suicidal ideation and attempts in adolescents and adults with BN in two population-based samples. | Community (Adult, Adolescents, All Genders) | Population | Prevalence of suicidal ideation and attempts |
| Cusack, et al. | 2021 | A network analysis of eating disorder symptoms and co-occurring alcohol misuse among heterosexual and sexual minority college women | 1027 | USA | to identify pathways, central symptoms, and bridge symptoms across alcohol misuse and eating disorder symptoms. | Community (Adult, Women) | Network Analysis | Network association |
| Dell'Osso, et al. | 2018 | Subthreshold Autism Spectrum Disorder in patients with Eating Disorders | 298 | Italy | to assess the presence of subthreshold ASD symptoms, by means of a recently validated instrument, in a sample of participants with EDs, particularly comparing participants with or without BE behaviours | College (Adult, All Genders) | Cross-Sectional | ASD symptoms in ED patients |
| Dinkler, et al | 2021 | Anorexia nervosa and autism: A prospective twin cohort study | 5987 | Sweden | to investigate autistic traits before and after the first diagnosis of AN. | Community (Children, All Genders) | Longitudinal (>5yr) | Autistic traits in children later diagnosed with AN |
| Dovey, et al. | 2019 | Eating behaviour, behavioural problems and sensory profiles of children with avoidant/restrictive food intake disorder (ARFID), autistic spectrum disorders or picky eating: Same or different? | 486 | London | to examine eating difficulties, behavioural problems and sensory hypersensitivity in ARFID children, relative to typically developing children with no reported feeding, mental or physical health problems, as well as children with ASD and picky eating. | Parents with or without children who have AFRID | Cross-Sectional | Eating difficulties, behavioural problems and sensory hypersensitivity |
| Duffy, et al. | 2018 | Prevalence of Self-Injurious Thoughts and Behaviours in Transgender Individuals With Eating Disorders: A National Study | 365,749 | USA | to examine prevalence of self-injurious thoughts and behaviours (SITBs) in transgender individuals with eating disorders, as compared to cisgender individuals with eating disorders and transgender individuals without eating disorders. | College (Adult, All Genders) | Cross-Sectional | Sociodemographic covariates, rates of past-year NSSI, suicidal ideation and suicide attempts, depression, ED dx |
| Eskild-Jensen, et al. | 2020 | Comorbid depression as a negative predictor of weight gain during treatment of anorexia nervosa: A systematic scoping review. | 15 studies | N/A | to investigate the influence of psychiatric comorbidity on weight gain during treatment of AN. |  | Scoping review | Psychiatric comorbidity as a predictor of weight gain |
| Espel-Huynh, et al. | 2019 | Mediating role of experiential avoidance in the relationship between anxiety sensitivity and eating disorder psychopathology: A clinical replication | 625 | USA | to examine whether anxiety sensitivity was associated with eating disorder (ED) symptom severity among patients with severe EDs, and to determine whether this relationship was mediated by experiential avoidance. | Inpatient (Adult, Adolescent, Women) | Cross-Sectional | Demographics, anxiety sensitivity, experiential avoidance and ED severity |
| Fenning & Hadas | 2010 | Suicidal behaviour and depression in adolescents with eating disorders | 46 | Israel | to examine suicidal behaviour and depression in adolescents with eating disorders, and to identify risk factors associated with suicidal ideation and attempted suicide. | Outpatient (Young People, Women, Dx with AN or BN) | Cross-Sectional | Sociodemographic covariates, ED psychopathology, suicide attempts and ideation, depression |
| Field, et al. | 2012 | Prospective Association of Common Eating Disorders and Adverse Outcomes | 8594 | UK & USA | to evaluate whether BN and subtypes of EDNOS are predictive of developing adverse outcomes. | Community (Children, Adolescents All Genders) | Longitudinal (>10yr) | Associations between EDs and adverse outcomes |
| Finzi-Dottan & Zubery | 2009 | The Role of Depression and Anxiety in Impulsive and Obsessive-Compulsive Behaviours Among Anorexic and Bulimic Patients | 169 | Israel | to assess the segregating link between impulsivity and obsessive-compulsiveness and types of eating disorder (bulimia nervosa vs. anorexia nervosa),and to examine the extent to which anxiety and depression account for the prediction of these dependant variables. | Outpatient (Adult, Women) | Cross-Sectional | Rates of OCD and impulsive behaviours |
| Forcano, et al. | 2009 | Suicide attempts in bulimia nervosa: personality and psychopathological correlates | 566 | Spain | to explore the prevalence of lifetime suicide attempts (SA) in women with bulimia nervosa (BN), and compared eating disorder symptoms, general psychopathology, impulsivity and personality between individuals who had and had not attempted suicide | Outpatient (Adult, Women) | Cross-Sectional | Sociodemographic covariates, ED Dx, lifetime suicide attempts, impulsivity, freq of binge eating and purging, personality and general psychopathology |
| Forcano, et al. | 2011 | Suicide attempts in anorexia nervosa subtypes | 214 | Spain | to explore the prevalence of lifetime suicide attempts in women with AN and compared those who had and had not attempted suicide on eating disorder symptoms, general psychopathology, and personality both relative to a healthy control group and then across AN subtypes. | Outpatient (Adult, All Genders, Dx with AN) | Cross-Sectional | Prevalence of suicide attempts, Depression symptoms, anxiety symptoms |
| Fornaro, et al. | 2021 | The prevalence, odds and predictors of lifespan comorbid eating disorder among people with a primary diagnosis of bipolar disorders, and vice-versa: Systematic review and meta-analysis. | 47 studies | N/A | to assess the prevalence and clinical correlates of BD⇌ED across the lifespan. |  | Systematic Review/ Meta-Analysis (combined) | Lifetime prevalence of comorbid EDs among people with bipolar disorder, and lifetime prevalence of comorbid bipolar disorder in EDs |
| Forrest, et al | 2021 | Suicide attempts among people with eating disorders and adverse childhood experiences: Results from a nationally representative sample of adults | 36146 | USA | to assess whether having ACEs and ED history augments risk for suicide attempts in a nationally representative sample of U.S. adults. | Community (Adult, All Genders) | Cross-Sectional | Adverse child experiences and suicide attempts for EDs |
| Fouladi, et al. | 2015 | Prevalence of Alcohol and Other Substance Use in Patients with Eating Disorders | 2966 | USA | to investigate any association between binge eating and purging and alcohol and substance use. | Outpatient (Adult, All Genders) | Cross-Sectional | Comorbidity of substance use disorders and EDs |
| Godart, et al. | 2015 | Mood disorders in eating disorder patients: prevalence and chronology of onset | 271 | France | to determine: (1) the frequency of mood disorders in all AN and BN subject groups, according to DSM-IV criteria; (2) whether mood disorders are significantly more frequent in ED patients than in the general population; (3) in cases with comorbidity, the relative chronology of onset of ED and mood disorder. | Outpatient (Young Adult, Women, Dx with AN or BN) | Cross-Sectional | Sociodemographics, ED Dx, major depressive episodes |
| Goel, et al. | 2018 | Correlates of Suicidal Ideation in College Women with Eating Disorders | 690 | USA | to identify the correlates of suicidal ideation (SI) in a large sample of college women with eating disorders (EDs). | College (Adult, Women) | Cross-Sectional | Sociodemographic covariates, ED psychopathology, general psychopathology, clinical impairment, BMI, suicidal ideation |
| Goldstein & Gvion | 2019 | Socio-demographic and psychological risk factors for suicidal behaviour among individuals with anorexia and bulimia nervosa: A systematic review | 38 | N/A | to analyse the relationship between AN and/or BN and suicidality (i.e. suicidal ideation or attempted and/or death by suicide) and the major risk factors for suicidal behaviour among AN and BN patients by synthesizing the qualitative data from relevant studies. | Community , Inpatient and Outpatient (Mixed Cohort, All Genders) | Review (Systematic) | Sociodemographic covariates, background factors, ED diagnosis, Pain tolerance, Interpersonal factors, Personality traits |
| Gosseaume, et al. | 2019 | Somatic complications and nutritional management of anorexia nervosa | N/A | N/A | to review severe undernutrition due to intake restriction and pathological associated behaviours such as potomania and vomiting causal impact on serious somatic complications such as cardiac and/or hepatic disturbances. |  | Review (Other) | Somatic complications of AN, Nutritional management of AN |
| Grenon et al | 2010 | Depressive Symptoms Are Associated with Medication Use and Lower Health-Related Quality of Life in Overweight Women with Binge Eating Disorder | 105 | Canada | to examine the relationships between depression, HRQOL, and health care utilization among treatment seeking women with BED. | Outpatient (Adult, Women) | Cross-Sectional | Demographics, health care utilisation, health care costs, depression score, and QoL |
| Grilo, et al. | 2013 | Psychiatric disorder co-morbidity and correlates in an ethnically diverse sample of obese patients with binge eating disorder in primary care settings | 142 | USA | to examine DSM-IV lifetime/current psychiatric disorder co-morbidity and correlates in ethnically-diverse obese patients with binge eating disorder (BED) seeking treatment for obesity and binge eating in primary care. | Outpatient (Young People, All Genders) | Cross-Sectional | Comorbidity and correlates of psychiatric diagnoses |
| Guilluame, et al. | 2011 | Characteristics of Suicide Attempts in Anorexia and Bulimia Nervosa: A Case-Control Study | 1563 | France | to explore whether the clinical characteristics of suicidal acts differ between suicide attempters with AN, BN or without an eating disorders (ED). | Inpatient (Adult, All Genders) Has been hospitalised for a suicide attempt. | Case control study | Suicide attempt, expectation of dying and suicide attempt severity |
| Harrop & Marlatt | 2010 | The comorbidity of substance use disorders and eating disorders in women: prevalence, etiology, and treatment | 13 studies | N/A | to address the prevalence and aetiology of this comorbidity (substance use and EDs) in women. |  | Review (Narrative) | Comorbidity of substance use disorders and EDs |
| Hazzard, et al. | 2021 | Treatment outcomes of psychotherapy for binge-eating disorder in a randomized controlled trial: Examining the roles of childhood abuse and post-traumatic stress disorder. | 112 | USA | to examine childhood abuse and post-traumatic stress disorder (PTSD) as predictors and moderators of binge-eating disorder (BED) treatment outcomes in a randomized controlled trial comparing Integrative Cognitive-Affective Therapy with cognitive-behavioural therapy administered using guided self-help. | Community, Outpatient (Adult, All Genders) | RCT | Childhood abuse, PTSD diagnosis, |
| Himmerich, et al. | 2019 | Psychiatric Comorbidity as a Risk Factor for the Mortality of People with Bulimia Nervosa | 1501 | UK | to investigate the influence of these psychiatric comorbidities on all-cause mortality with demographic and socioeconomic factors considered as confounders for people with BN | Inpatient (Adult, All Genders, Dx with BN) | Cross-Sectional | All-cause mortality, demographic and socioeconomic confounders |
| Huas, et al. | 2013 | Mortality and Its Predictors in Severe Bulimia Nervosa Patients | 258 | France | to improve knowledge on BN and mortality. | Inpatient (Adult, All Genders, Dx with BN) | Longitudinal (>10yr) | Deaths from suicide |
| Huke, et al. | 2013 | Autism Spectrum Disorders in Eating Disorder Populations: A Systematic Review | 8 studies | N/A | to investigate the prevalence of autism spectrum disorder in its entirety in eating disordered populations |  | Review (Systematic) | Prevalence rates of ASD in ED populations |
| Kaisari, et al. | 2017 | Attention Deficit Hyperactivity Disorder (ADHD) and disordered eating behaviour: A systematic review and a framework for future research | 75 studies | N/A | to systematically review the literature for an association between ADHD and disordered eating. |  | Review (Systematic) | Associations between ADHD and ED symptoms |
| Kambanis, et al. | 2019 | Prevalence and correlates of psychiatric comorbidities in children and adolescents with full and subthreshold avoidant/restrictive food intake disorder | 74 | USA | to characterize the current and lifetime prevalence of comorbid psychiatric diagnoses and suicidality in treatment- and non-treatment seeking individuals with full and subthreshold avoidant/restrictive food intake disorder (ARFID). | Community (Mixed Cohort, All Genders, Full or subthreshold ARFID) | Cross-Sectional | Prevalence and correlates of psychiatric comorbidities |
| Kerr-Gaffney, et al. | 2018 | Social Anxiety in the Eating Disorders: A Systematic Review and Meta-Analysis | 38 studies | N/A | to provide a qualitative summary of what is known about social anxiety (SA) in EDs, as well as to compare levels of SA in those with EDs and healthy controls. |  | Systematic Review/ Meta-Analysis (combined) | N/A |
| Koyanagi, et al. | 2016 | Psychotic-like experiences and disordered eating in the English general population | 7403 | UK | (a) to assess the association between depression (subtypes or severity) and psychotic experiences among adults, and (b) to assess whether depression with psychotic experiences are associated with more pronounced decrements in health status compared with depression alone. | Community (Adult, All Genders) | Cross-Sectional | Past 12-month occurrence of psychotic experiences and association with EDs |
| Kucukgoncu, et al. | 2014 | Clinical Features of Night Eating Syndrome Among Depressed Patients | 15 | Turkey | to investigate the frequency and clinical features of NES in a sample of patients with depression, focusing on the differences in clinical features (i.e., levels of depression, anxiety, obsession and compulsion, and sleep parameters) of those with and without NES and on the predictors of NES. | Outpatient (Adult, All Genders, Dx with Depression) | Cross-Sectional | Prevalence of night eating behaviours |
| Lundgren, et al. | 2012 | Lifetime Medical and Psychiatric Comorbidity of Night Eating Behaviour in the Swedish Twin Study of Adults: Genes and Environment (STAGE) | 627 | Sweden | to examine the lifetime medical and psychosocial comorbidity of two core features of NES, EH and NI | Community (Adult, All Genders) | Cross-Sectional | Prevalence of night eating behaviours |
| Mandelli, et al. | 2020 | Rates of comorbid obsessive-compulsive disorder in eating disorders: A meta-analysis of the literature. | 32 studies | N/A | to derive more consistent estimates of OCD in EDs, taking into account potential methodological and sampling confounding factors. |  | Meta-Analysis | Comorbidity |
| Martinussen, et al. | 2016 | The comorbidity of personality disorders in eating disorders: a meta-analysis | 87 studies | N/A | to summarize the proportion of comorbid personality disorders (PDs) in patients with anorexia (AN) and bulimia nervosa (BN), respectively, and examined possible moderating variables. |  | Meta-Analysis | Personality disorder comorbidity with EDs |
| Mayes, et al. | 2014 | Correlates of Suicide Ideation and Attempts in Children and Adolescents With Eating Disorders | 90 | USA | to determine correlates of suicide behaviour in children with eating disorders using multiple sleep, psychological, and demographic variables. | Parents or Carers of children with AN or BN (Women) | Cross-Sectional | Suicide ideation, attempts, sleep and psychological correlates, demographics |
| McAulay, et al. | 2021 | Eating disorder features in bipolar disorder: clinical implications | 73 | Australia | to identify which ED features are common in BD, and whether these relate to quality of life (QoL) impairment and body mass index (BMI). | Outpatient (Adult, All Genders) | Cross-Sectional | Health, ED features, emotion regulation, impulsivity and QoL |
| McDonald, et al. | 2019 | The comorbidity of eating disorders in bipolar disorder and associated clinical correlates characterised by emotional dysregulation and impulsivity: A systematic review | 9 studies | N/A | to compare the frequency of comorbid EDs to recent community prevalence rates. |  | Review (Systematic) | Comorbidity of ED across bipolar populations |
| McElory, et al. | 2016 | Clinical Features of Bipolar Spectrum With Binge Eating Behaviour | 1,114 | USA | to determine whether bipolar spectrum disorder with binge eating behaviour (BE) is an important clinical sub-phenotype. | Community (Adult, All Genders, Dx with Bipolar Disorder) | Cross-Sectional | Prevalence rates and correlates of binge eating in Bipolar Disorder |
| McElroy, et al. | 2016 | Prevalence and correlates of DSM-5 eating disorders in patients with bipolar disorder | 1092 | USA | to evaluate the prevalence and correlates of current DSM-5-defined BED, BN, and AN among patients with bipolar disorder | Outpatients with BN | Cross-Sectional | Comorbidity of EDs with bipolar disorder |
| McElroy, et al. | 2011 | Prevalence and correlates of eating disorders in 875 patients with bipolar disorder | 875 | USA | to determine the prevalence and clinical correlates of eating disorders in patients with bipolar disorder. | Outpatients (Adults) | Cross-Sectional | Clinical interview for BP and ED |
| Melo, et al. | 2018 | Night eating in bipolar disorder | 120 | Brazil | to identify NES in euthymic BD patients. | Outpatient (Adult, All Genders, Dx with Bipolar Disorder) | Cross-Sectional | Night eating symptoms, depression and manic symptoms, functioning, physical activity, anxiety, sleep quality, insomnia |
| Meule, et al. | 2014 | Emotional Eating Moderates the Relationship of Night Eating With Binge Eating and Body Mass | 729 | Germany | to test if there would be interactive effects of self-reported night and emotional eating when predicting binge eating frequency and BMI in a large student sample | Community (Adult, All Genders) | Cross-Sectional | Night eating symptoms, mood, binge eating episodes |
| Miotto, et al. | 2010 | Symptoms of psychosis in anorexia and bulimia nervosa | 748 | Italy | to investigate the comorbidity of psychosis and eating disorders | Group 1: Females 13-50yrs dx with an ED, Group 2: Female high school students | Cross-Sectional | Occurrence of symptoms of psychosis among clinical ED pop and non-clinical pop |
| Mitchell, et al. | 2021 | The impact of comorbid posttraumatic stress disorder on eating disorder treatment outcomes: Investigating the unified treatment model. | 2809 | USA | to examine the impact of PTSD diagnosis at admission on: ED symptom trajectories during treatment and after treatment, treatment drop-out, and clinically significant change in ED symptoms. | Inpatient (Adult, Adolescent, Women) | Repeated Measure (with follow-up) | Descriptives, ED symptoms and clinical improvement |
| Mitchell, et al. | 2021 | Posttraumatic stress disorder and eating disorders: maintaining mechanisms and treatment targets. | N/A | N/A | to review studies of the mechanisms associating trauma exposure and/or PTSD with EDs |  | Review (Narrative) | Mechanisms associated with trauma and ED comorbidity |
| Muehlenkamp, et al. | 2011 | Non-suicidal self-injury in eating disordered patients: A test of a conceptual model | 422 | Belgium | to test a theoretical model explaining the high co-occurrence of non-suicidal self-injury (NSSI) in eating disordered populations as resulting from childhood traumatic experiences, low self-esteem, psychopathology, dissociation, and body dissatisfaction was previously proposed but not empirically tested. | Inpatient (Young Adult, Women) | Modelling (Statistical) | Self harm, ED, psychopathology, childhood trauma, low self-esteem, dissociation, body dissatisfaction |
| Navarro-Haro, et al. | 2015 | The role of emotion regulation strategies and dissociation in non-suicidal self-injury for women with borderline personality disorder and comorbid eating disorder | 68 | Iceland | to examine the relationship of two emotion regulation strategies (i.e., expressive suppression and cognitive reappraisal) and dissociation with NSSI in women with BPD and comorbid ED. | Outpatient (Adult, All Genders), Clinic for personality disorders and EDs | Cross-Sectional | Emotion regulation strategies, dissociation and NSSI |
| Nazar, et al. | 2016 | The Risk of Eating Disorders Comorbid with Attention-Deficit/Hyperactivity Disorder: A Systematic Review and Meta-Analysis | 12 studies | N/A | to update and expand the previous systematic review and perform a meta-analysis of studies to investigate the risk of comorbidity with an ED (AN, BN or BED) in ADHD individuals or eating disorder symptoms in ADHD paediatric samples. |  | Systematic Review/ Meta-Analysis (combined) | Associations between ADHD and EDs |
| Olatunji, et al. | 2010 | Mediation of symptom changes during inpatient treatment for eating disorders: the role of obsessive compulsive features | 508 | USA | to examine the relative contributions of changes in obsessive compulsive symptoms among eating-disorder patients with and without obsessive compulsive disorder (OCD) to eating-disorder symptom improvement observed with inpatient treatment. | Inpatient (Young Adult, Women) | Cross-Sectional | Changes in ED and OCD symptoms |
| Olatunji, et al. | 2015 | Self-harm history predicts resistance to inpatient treatment of body shape aversion in women with eating disorders: The role of negative affect | 2061 | USA | to (a) evaluate the relationship between self-harm and changes in body dissatisfaction and drive for thinness in a large sample of patients who underwent inpatient treatment, and (b) to examine whether the relationship between self-harm and changes in body dissatisfaction and drive for thinness during inpatient treatment remains significant when controlling for change in negative affect during treatment. | Inpatient (Adult, All Genders) | Modelling (Statistical) | Impact of history of self-harm on changes in body dissatisfaction |
| Palmese, et al. | 2013 | Prevalence of Night Eating in Obese Individuals with Schizophrenia and Schizoaffective Disorder | 100 | USA | to establish the rates among individuals with schizophrenia and schizoaffective disorder. | Outpatient (Adult, All Genders, Dx with schizophrenia or schizoaffective disorder) | Cross-Sectional | Prevalence of night eating behaviours |
| Perez, et al. | 2018 | Non-suicidal Self-injury Differentiates Suicide Ideators and Attempters and Predicts Future Suicide Attempts in Patients with Eating Disorders | 238 | Spain | to identify psychopathological variables differentiating between suicide ideators, suicide attempters and patients without suicide ideation or attempts, and second to identify better predictors of suicide attempts longitudinally. | Outpatient (Adult, All Genders) | Repeated Measure (without follow-up) | SCID-5 for diagnosis, Suicide attempts, Self harm (Inventory of Statements about Self Injury), Hopelessness (Beck Hopelessness Scale), Suicidal Ideation (Beck Suicidal Ideation Scale), Borderline Personality Symptoms (Borderline Symptom List) |
| Perez, et al. | 2018 | Non-suicidal self-injury in patients with eating disorders: prevalence, forms, functions, and body image correlates | 226 | Spain | First, to explore the frequency, types, and functions of NSSI in a sample of female participants with EDs. Second, to explore differences in NSSI and body image depending on the ED restrictive-purgative subtype; and third, to explore differences in body dissatisfaction, body image orientation, and body investment in eating disorder patients without NSSI, with NSSI in their lifetime, and with NSSI in the previous year | Outpatients, Young Adult & Adult, Dx with ED) | Cross-Sectional | NSSI freq and function, body image distress, body image orientation and body image investment |
| Phillipou, et al. | 2019 | Direct comparisons of anorexia nervosa and body dysmorphic disorder: a systematic review | 15 studies | N/A | to review those studies that have directly compared groups of individuals with AN and BDD, to determine similarities and differences in presentation between the two conditions. | N/A | Review (Systematic) | Body dissatisfaction |
| Pila, et al. | 2019 | Reciprocal relations between dietary restraint and negative affect in adolescents receiving treatment for anorexia nervosa | 107 | Australia | to understand the reciprocal associations between higher and lower order dimensions of negative affect and dietary restraint were examined in adolescents undergoing treatment for AN. | Outpatient (Adolescents, Dx with AN) | Cross-Sectional | PANAS-X and EDE-Q (restraint) |
| Pisetsky, et al. | 2017 | A comparison of the frequency of familial suicide attempts across eating disorder diagnoses | 1870 | USA | to examine the prevalence of reported suicide attempts among family members of individuals with an eating disorder (ED). | Outpatient (Adult, All Genders) | Cross-Sectional | Family suicide attempts in individuals with BN |
| Pisetsky, et al. | 2015 | Depression and personality traits associated with emotion dysregulation: correlates of suicide attempts in women with bulimia nervosa | 337 | USA | (a) to determine the extent to which personality characteristics are associated with a lifetime history of a suicide attempt; (b) to examine which psychiatric comorbidities, including mood, substance use, and anxiety disorders, are associated with a lifetime history of a suicide attempt; and (c) to identify the unique effects of both personality and psychiatric comorbidity on a lifetime history of a suicide attempt among individuals with BN. | Community & Outpatient (Adults, Women, Dx with BN) | Cross-Sectional | Suicide attempts and comorbidities |
| Preti, et al. | 2011 | A Comprehensive Meta-Analysis of the Risk of Suicide in Eating Disorders | 59 studies | N/A | to analyse all the available information on death by suicide in eating disorders(EDs) to derive evidence on: i) the risk of suicide in patients diagnosed with EDs, using adequate comparison data; ii) whether the risk has decreased in the past years, taking into account the year of publication of the studies included in this paper. |  | Meta-Analysis | Suicides in ED patients |
| Ranta, et al. | 2017 | Social Phobia, Depression and Eating Disorders During Middle Adolescence: Longitudinal Associations and Treatment Seeking | 3278 | Finland | to examine these associations (social phobia, depression, ED) in a large-scale follow-up study among middle adolescents | Community (Adolescents, All Genders) | Repeated measure with follow up | Associations of comorbidities with ED treatment seeking |
| Rienecke, et al. | 2021 | Posttraumatic stress disorder symptoms and trauma-informed care in higher levels of care for eating disorders | 613 | USA | to examine the prevalence and trajectory of posttraumatic stress disorder (PTSD) symptoms among patients with eating disorders (EDs) in higher level of ED care with trauma-informed components, but without a formal evidence-based trauma intervention. | Inpatient, Outpatient (Adult, All Genders) | Repeated Measure (without follow-up) | Change in PTSD symptoms |
| Riley, et al. | 2016 | Non-suicidal self-injury as a risk factor for purging onset: Negatively reinforced behaviours that reduce emotional distress | 1158 | USA | to test two predictive pathways using a prospective design; specifically, engagement in NSSI would predict the college onset of purging, and purging would predict the onset of NSSI. | College (Adult, Women) | Repeated Measure (without follow-up) | Sociodemographics, Deliberate self-harm, ED psychopathology (EDE-Q) |
| Root, et al. | 2010 | Patterns of co-morbidity of eating disorders and substance use in Swedish females | 13,297 | Sweden | to examine the association between EDs and SU in a large population-based sample. | Community (Adult, All Genders) | Population | Demographics, prevalence rates |
| Rowe, et al. | 2010 | Complex Personality Disorder in Bulimia Nervosa | 134 | New Zealand | to evaluate the ability of Tyrer's dimensional approach to predict pathology and outcome in a sample of women with BN participating in a randomized controlled trial of cognitive behaviour therapy. | Outpatient (Adult, Women, Dx with BN) | Repeated Measure (with follow-up) | Axis 1 comorbidity and psychopathology in personality disorder groups |
| Runfola, **et** al. | 2014 | Self-image and suicide in a Swedish national eating disorders clinical register | 2269 | Sweden | to examine the relation between self-image (assessed using the Structural Analysis of Social Behaviour) and suicide attempts/completions in women with anorexia nervosa-restricting type (ANR), anorexia nervosa-binge/purge type (ANBP), bulimia nervosa (BN), binge eating disorder (BED), and eating disorder not otherwise specified (EDNOS); and to assess whether these self-image variables add unique predictive value to suicide when considering other baseline predictors. | Outpatient (Adult, Women) | Prospective | Data on age, body mass index, eating disorder severity (Eating Disorder Examination-Questionnaire scores), psychiatric comorbidity, global assessment of functioning, and self-image |
| Sagiv & Gvion | 2020 | A multi factorial model of self-harm behaviours in Anorexia-nervosa and Bulimia-nervosa | 93 | Israel | to explore the mediating role of depression, LA, trait impulsivity, and rumination in the relationships between EDs and SI, SA, and NSSI. | Inpatient (Adult, All Genders, Dx with AN or BN) | Cross-Sectional | Sociodemographic covariates, ED dx, suicide attempts (SBQ-R), self-harm (DSHI), Depression (PHQ-9), impulsivity, loss aversion and binge frequency |
| Schnicker, et al. | 2013 | Drop-out and treatment outcome of outpatient cognitive-behavioural therapy for anorexia nervosa and bulimia nervosa | 104 | Germany | to find out what proportion of subjects with an eating disorder (AN, BN) prematurely terminated their participation, in a naturalistic therapeutic study. Secondly, to find out how subjects who regularly completed the therapy (completers) differed from those who terminated it prematurely (drop-outs). Thirdly, to find out what effect the therapy had on both the completers and drop-outs. | Outpatient (Young Adult, Women, Dx with AN or BN) | Cross-Sectional | ED Dx and symptoms (EDI-2), BMI, Depression (BDI & BSI), therapy response |
| Seitz, B. et al. | 2013 | The Role of Impulsivity, Inattention and Comorbid ADHD in Patients with Bulimia Nervosa | 97 | Germany | to examine patients with BN and healthy controls (HC) to determine the severity of their impulsivity and inattention, as well as the rate of childhood and current ADHD symptoms | Inpatient, Outpatient (Adult, Women, Dx with BN) | Cross-Sectional | Prevalence of ADHD in ED patients with BN. Associations between BN and ADHD symptoms |
| Seixas, et al. | 2012 | Prevalence and clinical impact of eating disorders in bipolar patients | 356 | Brazil | to study the impact of eating disorders (EDs) on the severity of bipolar disorder | Inpatient (Adult, All Genders, Dx with Bipolar Disorder) |  | Prevalence of ED symptoms in patients with Bipolar Disorder |
| Selby, et al. | 2010 | Habitual Starvation and Provocative Behaviours: Two Potential Routes to Extreme Suicidal Behaviour in Anorexia Nervosa | 1036 | USA | to explore whether repetitive exposure to painful and destructive behaviours such as vomiting, laxative use, and non-suicidal self-injury (NSSI) was a mechanism that linked AN-binge-purging (ANBP) subtype, as opposed to AN-restricting subtype (ANR), to extreme suicidal behaviour. | Clinical Settings (Outpatient and Inpatient, Mixed Cohort, All Genders). Lifetime diagnosis of AN by 45 | Cross-Sectional | Sociodemographic covariates, SCID for ED dx, EATATE (personality traits, provocative behaviours), suicidal behaviour (number of attempts, lethality of most serious attempt, intent to die), co-occurring psychopathology. |
| Simpson, et al. | 2013 | Treatment of obsessive-compulsive disorder complicated by comorbid eating disorders | 56 | USA | to examine the naturalistic outcome of patients with both disorders (OCD and EDs), who received a multimodal treatment program designed to address both problems simultaneously. | Inpatient (Adult, All Genders) | Repeated Measure (without follow-up) | Changes in ED and OCD symptoms and depression severity |
| Smith, et al. | 2017 | Emotional reactivity in a clinical sample of patients with eating disorders and non-suicidal self-injury | 648 | USA | to address these gaps by examining emotional reactivity and other relevant domains in a large clinical sample of patients categorized into ED-only, NSSI-only, and co-occurring ED and NSSI groups | Inpatient, Day program and intensive outpatient for ED and/or NSSI | Cross-Sectional | Emotional reactivity, QoL, NSSI frequency, EDE-Q |
| Spettigue, et al. | 2020 | Binge eating and social anxiety in treatment-seeking adolescents with eating disorders or severe obesity. | 490 | Canada | to explore the association between social anxiety and binge eating and compare prevalence rates of social anxiety between youth with severe obesity or EDs who did or did not engage in binge eating. | Outpatient (Young People, All Genders) | Cross-Sectional | Social anxiety, eating behaviours |
| Spiegel, et al. | 2021 | Emotional abuse interacts with borderline personality in adolescent inpatients with binge-purging eating disorders. | 128 | Germany | to investigate the correlations between childhood emotional neglect (CEN), childhood emotional abuse (CEA), and obsessive-compulsive and borderline personality styles in female adolescent inpatients with eating disorders (EDs). | Inpatient (Young People, All Genders) | Cross-Sectional | Associations between childhood abuse and neglect on personality styles in inpatients diagnosed with ED |
| Striegel-Moore, et al. | 2010 | Nocturnal Eating: Association with Binge Eating, Obesity, and Psychological Distress | 285 | USA | to examine clinical correlates of nocturnal eating, a core behavioural symptom of night eating syndrome. | Community (Adult, Women) | Cross-Sectional | Nocturnal eating episodes, ED symptoms, BMI, psychosocial measures |
| Svedlund, et al. | 2018 | Are treatment results for eating disorders affected by ADHD symptoms? A one-year follow-up of adult females | 443 | Sweden | to explore the influence of self-reported Attention Deficit Hyperactivity Disorder (ADHD) symptoms on recovery rate at 1-year follow-up in an unselected group of patients in a specialized eating disorder (ED) clinic. | Outpatient (Adult, Women) | Repeated Measure (with follow-up) | Impact of ADHD symptoms on ED recovery |
| Svedlund, et al. | 2017 | Symptoms of Attention Deficit Hyperactivity Disorder (ADHD) Among Adult Eating Disorder Patients | 1094 | Sweden | to explore the prevalence and types of self-reported ADHD symptoms in an unselected group of eating disorder patients assessed in a specialized eating disorder clinic. | Outpatient (Adult, Women) | Cross-Sectional | Prevalence and types of ADHD symptoms in ED population |
| Swinbourne, et al. | 2012 | The comorbidity between eating disorders and anxiety disorders: Prevalence in an eating disorder sample and anxiety disorder sample. | 152 | Australia | to investigate the prevalence of comorbid eating and anxiety disorders in women presenting for inpatient and outpatient treatment of an eating disorder and women presenting for outpatient treatment of an anxiety disorder. | Inpatient (Adult, Women) | Cross-Sectional | Anxiety disorder and prevalence rates |
| Thiebaut, et al. | 2019 | Impact of bipolar disorder on eating disorders severity in real-life settings | 261 | France | to investigate the association between a dual diagnosis and severity in terms of clinical, neuropsychological dimensions and daily functioning. | Inpatient (Adult, All Genders) | Cross-Sectional | Bipolar dx, ED Dx and symptoms, psychiatric functioning, neurocognitive dimensions |
| Thiebaut, et al. | 2018 | Crossed prevalence results between subtypes of eating disorder and bipolar disorder: A systematic review of the literature | 79 studies | N/A | to assess the association between sub-types of bipolar disorder (BD) (types I and II) and AN, BN, and BED as well as their relative order of occurrence. |  | Review (Systematic) | Comorbidity of EDs with bipolar disorder |
| Thornton et al. | 2017 | Binge-eating disorder in the Swedish national registers: Somatic comorbidity | 850 | Sweden | to evaluate associations between binge‐eating disorder (BED) and somatic illnesses and determine whether medical comorbidities are more common in individuals who present with BED and comorbid obesity. |  | Population | Demographics, prevalence rates |
| Thornton, et al. | 2016 | Anorexia Nervosa, Major Depression and Suicide Attempts: Shared Genetic Factors | 6899 | USA | to evaluate the extent to which genetic and environmental factors influenced anorexia nervosa (AN), major depressive disorder (MDD), and suicide attempts (SA) | Community (Adult, Women) | Cross-Sectional | Diagnosis of AN, Depression, suicide attempts |
| Thornton, et al. | 2011 | Anorexia nervosa and generalized anxiety disorder: further explorations of the relation between anxiety and body mass index | 6085 | USA | 1) to document comorbidity patterns of AN and GAD in a large population-based sample of Swedish twins; 2) to explore the relation among AN, GAD, and BMI; and 3) to assess whether fasting and excessive exercise are more commonly endorsed by individuals with AN and GAD than those with AN or GAD only or the referent group of individuals with neither AN nor GAD. | Community (Adult, Women) | Cross-Sectional | AN Diagnosis, Generalised Anxiety Diagnosis, BMI, Fasting and excessive exercise |
| Touchette, et al. | 2011 | Subclinical eating disorders and their comorbidity with mood and anxiety disorders in adolescent girls | 833 | Canada | to assess the prevalence of subclinical eating disorders, major depression, dysthymia, separation anxiety, and generalized anxiety disorders. | Community (Adolescents, Girls) | Cross-Sectional | Demographics, prevalence of subclinical EDs and psychiatric comorbidities |
| Udo, et al. | 2019 | Suicide attempts in US adults with lifetime DSM-5 eating disorders | 36171 | USA | to (1) obtain the prevalence estimates of SA in US adults with lifetime DSM-5 ED diagnoses, (2) examine sociodemographic, clinical, and psychiatric correlates of SA in EDs, and (3) examine temporary relationships between DSM-5 ED diagnoses and first SA. | Community (Adult, All Genders) | Cross-Sectional | Sociodemographic covariates, diagnostic assessment of EDs and other disorders (AUDADIS-5), suicide attempts (SAs) |
| Ulfvebrand, et al. | 2015 | Psychiatric comorbidity in women and men with eating disorders results from a large clinical database | 11,588 | Sweden | to investigate psychiatric DSM-IV Axis I comorbidity in a large sample of adult patients, both males and females, with the whole spectrum of DSM-IV ED diagnoses. | Outpatient (Adult, All Genders) | Cross-Sectional | Psychiatric diagnosis comorbidities |
| Vanzhula, et al. | 2021 | Perfectionism and Difficulty Controlling Thoughts Bridge Eating Disorder and Obsessive-Compulsive Disorder Symptoms: A Network Analysis | 1619 | USA | to (1) identify bridge pathways in an ED-OCD comorbidity network and (2) test if perfectionism symptoms bridge between ED-OCD symptoms in a combined network model including ED, OCD, and Perfectionism symptoms. | Community (Adult, All Genders) | Network Analysis | Intrusive cognitions and maladaptive perfectionism |
| Voderholzer, et al. | 2015 | Association between depressive symptoms, weight and treatment outcome in a very large anorexia nervosa sample | 418 | Germany | to investigate whether the degree to which patients were underweight and level of depression were associated. Secondly, to examine whether the level of depression before treatment as well as changes in depressive symptoms, among other possible variables, predicted treatment outcome in AN. | Inpatient (Adult, Women, Dx with AN, treatment seeking) | Repeated Measure (without follow-up) | Sociodemographic covariates, Depression (BDI), BMI, Comorbid psychiatric disorders |
| Vrabel et al | 2010 | Five-Year Prospective Study of Personality Disorders in Adults with Longstanding Eating Disorders | 74 | Norway | to (1) report the occurrence of personality disorders in adults with eating disorders during inpatient treatment, and at 1-, 2-, and 5-year follow-up, (2) compare the changes of personality disorders in anorexia nervosa, bulimia nervosa, and eating disorder not otherwise specified, and (3) investigate if recovered patients had lower frequency of personality disorders. | Inpatient (Adult, All Genders) | Longitudinal (<5yr) | Demographics, personality disorder comorbidity |
| Westwood, et al. | 2017 | Clinical evaluation of autistic symptoms in women with anorexia nervosa | 60 | UK | to use a standardised, clinical assessment of ASD, the Autism Diagnostic Observation Schedule, 2nd Edition (ADOS-2), to investigate the presence of autistic symptoms in a cross-sectional sample of women with AN. | Inpatient, Outpatient (Adult, Women) | Cross-Sectional | Symptoms of ASD in women with AN, association with psychiatric symptoms |
| Yao, et al. | 2016 | Familial Liability for Eating Disorders and Suicide Attempts: Evidence From a Population Registry in Sweden | 2268786 | Sweden | to examine the association between eating disorders and suicide attempts and whether familial risk factors contribute to the association. | Community (Children, All Genders) | Longitudinal (>5yr) | association between eating disorders and death by suicide |
| Zhang, et al. | 2020 | Development of Disordered Eating Behaviours and Comorbid Depressive Symptoms in Adolescence: Neural and Psychopathological Predictors. | 1386 | Europe | to investigate early psychopathological and neuroanatomical risk 7factors for the development of DEBs and comorbid mental health problems. |  | Longitudinal (>5yr) | Development of ED behaviours and associated mental health problems, changes in voxel-based morphometry and psychopathological differences |
| Ziobrowski & Brewerton | 2017 | Associations between ADHD and Eating Disorders in Relation to Comorbid Psychiatric Disorders in a Nationally Representative Sample | 4719 | USA | to examine whether previously observed associations of ADHD with eating disorders are particularly attributable to other underlying psychopathology | Community (Adult, All Genders) | Cross-Sectional | Associations between ADHD and EDs |

**Table 4.** Studies regarding medical comorbidities included in the rapid review

| **Medical Comorbidities** | | | | | | | | |
| --- | --- | --- | --- | --- | --- | --- | --- | --- |
| **Author** | **Year** | **Title** | **N** | **Country** | **Aim** | **Population** | **Design** | **Outcome** |
| Abraham & Kellow | 2011 | Exploring eating disorder quality of life and functional gastrointestinal disorders among eating disorder patients | 160 | Australia | to explore the relationship between the QOL of ED patients and FGIDs. | Inpatient (Adult, Women) | Cross-Sectional | Quality of life, bowel symptoms, ED symptoms |
| Agostino, et al. | 2013 | Shifting Paradigms: Continuous Nasogastric Feeding with High Caloric Intakes in Anorexia Nervosa | 165 | Canada | to demonstrate a difference in length of admission between adolescent eating disorder patients treated with short-term continuous NG feeding at high caloric intakes compared with those who were managed with standard lower calorie bolus meal therapy. | Inpatient (Young People, All Genders, Dx with AN) | Cross-Sectional | Length of stay, weight gain, feeding method |
| Algars, et al. | 2014 | Binge eating and menstrual dysfunction | 11503 | Sweden | to investigate the association between lifetime binge eating and menstrual dysfunction in a large sample of female twins. | Community (Adult, All Genders) | Cross-Sectional | BMI, Binge eating behaviours, compensatory behaviours, menstrual dysfunction |
| Avila, et al. | 2019 | Eating disorders in adolescents with chronic gastrointestinal and endocrine diseases | N/A | N/A | to describe what is known about eating disorders in adolescents with chronic gastrointestinal and endocrine diseases, focusing on coeliac disease, inflammatory bowel disease, diabetes, and thyroid disorders. | Adolescents | Review (Systematic) | Prevalence and onset of gastrointestinal and endocrine diseases, associations with EDs |
| Becker, et al. | 2021 | Ghrelin and PYY in low-weight females with avoidant/restrictive food intake disorder compared to anorexia nervosa and healthy controls | 94 | USA | to compare fasting and postprandial concentrations of total ghrelin and total PYY in adolescent/young adult females with low-weight ARFID, AN and atypical AN, and HC. | Outpatient (Adult, Women, Dx with AN or ARFID) | Cross-Sectional | BMI, Ghrelin and PYY |
| Bemer, et al. | 2021 | Bone mineral density at extremely low weight in patients with anorexia nervosa. | 97 | France | to assess prevalence and clinic-biological correlates of low BMD and fractures in extremely undernourished inpatients with AN. | Inpatient (Adult, Women) | Cross-Sectional | Factors associated with low BMD and fractures |
| Benini, et al | 2010 | Oesophageal motility and symptoms in restricting and binge-eating/purging anorexia | 23 | Italy | to clarify whether oesophageal symptoms in AN are associated with motility disorders, with different forms of the disease, and whether they respond to nutritional rehabilitation. | Inpatient (Adult, All Genders) | Repeated Measure (without follow-up) | ED psychopathology, distress, oesophageal, gastric and colonic symptoms |
| Bloch, et al. | 2012 | Dehydroepiandrosterone treatment effects on weight, bone density, bone metabolism and mood in women suffering from anorexia nervosa-a pilot study | 26 | Israel | to assess the effects of DHEA treatment on weight and bone metabolism in this population. Our secondary goal was to assess the effects of DHEA administration on primary anorexic symptoms and mood | Outpatient (Adult, Women, Dx with AN) | RCT | Outcome measures were bone mineral density (BMD) and bone mineral content (BMC) measured by dual energy X-ray absorptiometry (DXA) and metabolism indexes, steroid hormones, and mood and eating disorder symptoms measured at baseline and at the 3 and 6 months follow-up visits. |
| Bouquegneau, et al | 2012 | Anorexia Nervosa and the Kidney | N/A | N/A | to discuss kidney diseases and kidney-associated conditions that occur in individuals with anorexia nervosa, summarizing many of the challenges in treating patients with this disease. | Focussed on AN | Review (Narrative) | Kidney disease and related conditions, Treatment challenges |
| Brewster, et al. | 2015 | Risk of oesophageal cancer among patients previously hospitalised with eating disorder | 3617 | Scotland | to investigate the risk of oesophageal cancer among patients with a prior history of hospitalisation with eating disorder. | Aged between 10-60 years | Cross-Sectional | Oesophageal cancer |
| Brown, et al. | 2015 | Predictors of Hypophosphatemia during Refeeding of Patients with Severe Anorexia Nervosa | 123 | USA | to identify clinically useful markers that may predict the development of or protection from hypophosphatemia during refeeding. | Inpatient (Adult, All Genders, Dx with AN) | Cross-Sectional | Prevalence of hypophosphatemia, sociodemographic, anthropometric measurements and caloric intake |
| Bulik, et al. | 2009 | Birth Outcomes in Women with Eating Disorders in the Norwegian Mother and Child Cohort Study (MoBa) | 35929 | USA | to explore the impact of eating disorders on birth outcomes in the Norwegian Mother and Child Cohort Study. | Pregnant women | Repeated Measure (without follow-up) | Pregnancy and birth information, health of neonate, ED symptoms and diagnosis |
| Cass, et al. | 2020 | Medical Complications of Anorexia Nervosa | N/A | N/A | to describe the medical complications of anorexia nervosa (AN) to enable a consult-liaison psychiatrist to be familiar with these complications when involved with the care of a hospitalized patient with AN. | Inpatient (Adult, All Genders, Dx with AN) | Review (Systematic) | How consult-liaison psychiatrists can help hospitalised AN patient |
| Chaer, et al. | 2020 | Fertility and Reproduction after Recovery from Anorexia Nervosa: A Systematic Review and Meta-Analysis of Long-Term Follow-Up Studies | 5 studies | N/A | to conduct a systematic review of the major reproductive health outcomes in females after recovery from AN. | | Systematic Review/ Meta-Analysis (combined) | Normalisation of reproductive functions (fertility, pregnancy and childbirth rates) following AN treatment |
| Cioffi, et al. | 2021 | The incidence of the refeeding syndrome. A systematic review and meta-analyses of literature | 35 studies | Italy | to estimate the incidence of RFS in adults by considering the definition used by the authors as well as the recent criteria proposed by the American Society of Parenteral and Enteral Nutrition (ASPEN) consensus. | | Systematic Review/ Meta-Analysis (combined) | Incidence of refeeding syndrome |
| Conviser, et al. | 2014 | Oral care behaviour after purging in a sample of women with bulimia nervosa | 201 | USA | to understand patients with BN oral health behaviours after purging and their perspectives on barriers to patient-initiated discussion of EDs with OHPs. | Outpatient (Adult, Women, Dx with BN) | Cross-Sectional | Oral care behaviours after urging, dental problems and barriers to communication |
| Davies, et al. | 2016 | The medical risks of severe anorexia nervosa during initial re-feeding and medical stabilisation | 65 | UK | to describe the medical risk profile, management and outcomes of a cohort of patients with severe anorexia nervosa (sAN) during medical stabilisation treatment. | Inpatient (Adult, Women, Dx with severe AN) | Case Series | Medical complications |
| DiVasta, et al. | 2013 | Does Hormone Replacement Normalize Bone Geometry in Adolescents With Anorexia Nervosa? | 80 | USA | to investigate the effects of oral DHEA + combined oral contraceptive (COC) versus placebo on changes in bone geometry in young women with AN | Inpatient (Young People, Women, Dx with AN) | RCT | Areal BMD and BMC of whole body, lumbar spine and total hip measured by DXA |
| Fazeli, et al. | 2014 | Teriparatide Increases Bone Formation and Bone Mineral Density in Adult Women With Anorexia Nervosa | 21 studies | N/A | to investigate the effect of teriparatide (TPT; human PTH[1–34]), an anabolic agent, on low bone mass in women with AN. | Outpatient (Adult, Women, Dx with AN) | RCT | Change in BMD of the spine and hip |
| Gallant et al. | 2014 | Night Eating Behaviour and Metabolic Heath in Mothers and Fathers Enrolled in the QUALITY Cohort Study | 310 | Canada | to examine the metabolic health correlates of night eating syndrome in adults enrolled in the QUALITY cohort study. | Community (Children, All Genders) | Cross-Sectional | Night eating behaviour, anthropometric measures, metabolic syndrome |
| Gallant, et al. | 2015 | Night-eating Symptoms and 2-year Weight Change in Parents Enrolled in the QUALITY Cohort | 388 | Canada | to test the hypothesis that symptoms commonly associated with night-eating syndrome are related to measures of weight gain in adults. | Community (Parents, All Genders) | Cross-Sectional | Night eating symptoms, BMI, waist circumference |
| Garber, et al. | 2013 | Higher Calorie Diets Increase Rate of Weight Gain and Shorten Hospital Stay in Hospitalized Adolescents with Anorexia Nervosa | 56 | USA | to compare higher and lower calorie diets during refeeding in moderately malnourished adolescents hospitalized with AN. | Inpatient (Young People, Women, Dx with AN) | Quasi-experimental (intervention) | Weight, heart rate, temperature, hydration markers and serum phosphorus. |
| Gentile, et al. | 2010 | Specialized refeeding treatment for anorexia nervosa patients suffering from extreme undernutrition | 33 | Italy | to describe our experience with the gradual correction of extreme undernutrition in AN patients, in order to reduce, besides morbidity and mortality, also the potential side-effects of refeeding. | Inpatient (Adult, Women, Dx with AN) | Cross-Sectional | Mean BMI, refeeding approach, refeeding complications |
| Gibson, et al. | 2021 | The intersect of gastrointestinal symptoms and malnutrition associated with anorexia nervosa and avoidant/restrictive food intake disorder: Functional or pathophysiologic? -A systematic review. | 146 studies | N/A | to better understand how these physiologic changes of malnutrition of the oesophagus, stomach, intestines, and pancreas contribute toward the reported GI symptoms, as well as better understand how celiac disease, inflammatory bowel disease, pelvic floor dysfunction, and Ehlers-Danlos syndrome contribute toward disordered eating. | | Review (Systematic) | Pathophysiologic changes of the oesophagus, stomach and intestines in patients with restrictive EDs |
| Giollo, et al. | 2017 | Vitamin D levels strongly influence bone mineral density and bone turnover markers during weight gain in female patients with anorexia nervosa | 91 | Italy | to investigate a potential role for vitamin D status on bone mineral density (BMD) during weight gain in patients with anorexia nervosa (AN). | Inpatient (Adult, adolescent, Women, Dx with AN) | Repeated Measure (without follow-up) | Spine and hip BMD |
| Giovinazzo, et al. | 2018 | Anorexia nervosa and heart disease: a systematic review | 88 studies | N/A | to review the cardiac complications of AN and give practical indications for their recognition and management. | | Review (Systematic) | Conduction disease, ventricular repolarization, arrhythmias, structural heart disease, Ischemic heart disease, atherosclerosis, coronary artery disease, refeeding syndrome |
| Golden, et al. | 2013 | Higher Caloric Intake in Hospitalized Adolescents With Anorexia Nervosa Is Associated With Reduced Length of Stay and No Increased Rate of Refeeding Syndrome | 310 | USA | to determine the effect of higher caloric intake on weight gain, length of stay (LOS), and incidence of hypophosphatemia, hypomagnesemia, and hypokalaemia in adolescents hospitalized with anorexia nervosa. | Inpatient (Young People, All Genders, Dx with AN) | Cross-Sectional | Incidence of hypophosphatemia (≤3.0 mg/dL), hypomagnesemia (≤1.7 mg/dL), and hypokalaemia (≤3.5 mEq/L), and daily change in percent median body mass index (BMI) (%mBMI) |
| Hale & Logomarsino | 2019 | The Use of Enteral Nutrition in the Treatment of Eating Disorders: A Systematic Review | 22 | N/A | to investigate the efficacy of EN in the treatment of eating disorders and make recommendations for clinical practice and future research. | Inpatient (All ages, All Genders, Dx with AN or BN) | Review (Systematic) | Adverse events, feeding modality, type of enteral formula used, duration of enteral nutrition, length of follow up |
| Hanachi, et al. | 2013 | Hypertransaminasemia in severely malnourished adult anorexia nervosa patients: Risk factors and evolution under enteral nutrition | 126 | France | to identify prevalence and risk factors of hyperaminotransferasemia in an adult cohort of AN patients and to describe evolution during nutritional rehabilitation with enteral nutrition for a period of 4 weeks | Inpatient (Adult, All Genders, Dx with AN) | Cross-Sectional | Risk factors for hypertransaminasemia |
| Hemmingsen, et al. | 2021 | Cognitive improvement following weight gain in patients with anorexia nervosa: A systematic review | 24 studies | N/A | to examine whether cognitive functions improve after weight gain in patients with AN. | | Review (Systematic) | Change in cognitive functions after weight gain |
| Hermont, et al. | 2013 | Eating Disorder Risk Behaviour and Dental Implications Among Adolescents | 1203 | Brazil | to compare the occurrence of tooth erosion (TE) and dental caries (DC) in adolescents with and without risk behaviour for eating disorders (EDs). | Community (Adolescents, Girls) | Cross-Sectional | Risk for ED, dental health |
| Hermont, et al. | 2014 | Tooth Erosion and Eating Disorders: A Systematic Review and Meta-Analysis | 23 papers | N/A | to search for scientific evidence regarding the following clinical question: Do eating disorders increase the risk of tooth erosion? | | Systematic Review/ Meta-Analysis (combined) | Tooth erosion in controls vs ED patients |
| Hofman, et al. | 2009 | Prevalence and clinical determinants of low bone mineral density in anorexia nervosa | 286 | Netherlands | to determine the prevalence of low bone mass in anorexia nervosa (AN) and the association with clinical parameters. | Inpatient (Adult, Women, Dx with AN) | Cross-Sectional | BMD as measured by DXA |
| Hood, et al. | 2014 | Night Eating in Patients with Type 2 Diabetes. Associations With Glycaemic Control, Eating Patterns, Sleep, and Mood | 194 | USA | to understand night eating and Night Eating Syndrome (NES) in patients with prevalent metabolic conditions, such as diabetes | Community (Adult, All Genders, Dx with T2 Diabetes) | Cross-Sectional | Night eating symptoms, sleep, glycaemic control data, haemoglobin A1c |
| Hung, et al. | 2021 | Anorexia Nervosa and Osteoporosis | N/A | N/A | to explore the prevalence and pathophysiology of osteoporosis and fractures in patients with AN, as well as published observations and studies on treatment | Patients with AN | Review (Narrative) | Factors implicated in AN-related bone disease, treatment of AN related-bone disease |
| Jaworski, et al. | 2018 | A Ten-Year Longitudinal Study of Prevalence of Eating Disorders in the General Polish Type 2 Diabetes Population | N/A | Poland | to evaluate the prevalence of EDs in T2DM among Polish patients whose treatment was financed by the Polish National Health Fund-NFZ, in the years 2008–2017. | Inpatient (All ages All Genders, Dx with T2 Diabetes) | Longitudinal (>10yr) | Prevalence of EDs in population diagnosed with Type 2 diabetes |
| John, et al. | 2021 | clinical management and mortality risk in those with eating disorders and self-harm: e-cohort study using the SAIL databank | 82627 | UK | to explore healthcare utilisation and mortality in those with a record of: self-harm only; eating disorders only; and both co-occurring. | | Cross-Sectional | Healthcare contacts, mortality |
| Kalla, et al. | 2017 | Gender and age differences in cardiovascular complications in anorexia patients | 9502 | USA | to assess cardiac manifestation of AN based on a large, national database and to assess gender and age differences. | Outpatient (Adult, All Genders, Dx with AN) | Cross-Sectional | Demographics, in hospital clinical outcomes, repolarization and conduction abnormalities, structural complications |
| Kappou, et al. | 2021 | Neuroimaging Findings in Adolescents and Young Adults with Anorexia Nervosa: A Systematic Review. | 33 studies | N/A | to examine neuroimaging findings in adolescents and young adults up to 24 years old, in order to explore alterations associated with disease pathophysiology. | | Review (Systematic) | Changes in structural and functional brain images |
| Karamanis, et al. | 2014 | Cancer incidence and mortality patterns in women with anorexia nervosa | 6009 | Sweden | to assess cancer incidence and mortality in women with AN. | Inpatient (Adult, children and young people, Female, Dx with AN) | Repeated Measure (with follow-up) | Sociodemographic covariates, cancer diagnosis and type, cancer incidence |
| Kisely, et al. | 2015 | Association Between Poor Oral Health and Eating Disorders: Systematic Review and Meta-Analysis | 10 studies | N/A | to determine the association between eating disorder and poor oral health, including any difference between patients with and without self-induced vomiting. | | Systematic Review/ Meta-Analysis (combined) | Oral health, ED Dx |
| Kolstad, et al. | 2015 | Epilepsy and eating disorders during pregnancy: Prevalence, complications and birth outcome | 107919 | Norway | to investigate the prevalence of eating disorders and its relation to pregnancy and delivery complications in childbearing women with epilepsy (WWE). | Pregnant women | Cross-Sectional | ED behaviours, epilepsy diagnosis, hypertension, diabetes, preeclampsia, peripartum depression and/or anxiety |
| Lebow & Sim | 2013 | The influence of estrogen therapies on bone mineral density in premenopausal women with anorexia nervosa and amenorrhea | | N/A | to examine the effectiveness of estrogen therapies on bone mineral density in women with amenorrhea | Dx with AN | Review (Narrative) | Effectiveness of estrogen therapies on BMD |
| Leclarc, et al. | 2013 | Evaluation of a nutrition rehabilitation protocol in hospitalized adolescents with restrictive eating disorders | 29 | Canada | to examine weight gain, prevalence of refeeding syndrome, and nutritional composition of the diet in hospitalized adolescents with anorexia nervosa (AN) and eating disorder not otherwise specified (EDNOS), restrictive type, on a structured nutrition rehabilitation protocol (NRP). | Inpatient (Young People, All Genders, Dx with AN) | Cross-Sectional | Weight gain, demographics, prevalence of refeeding syndrome |
| Leitner, et al. | 2016 | Prophylactic Phosphate Supplementation for the Inpatient Treatment of Restrictive Eating Disorders | 70 | Canada | to determine the safety and efficacy of a refeeding strategy that incorporates prophylactic phosphate supplementation to prevent RH. | Inpatient (Young People, All Genders, Dx with AN) | Cross-Sectional | %mBMI, prevalence of refeeding hypophosphatemia |
| Linna, et al | 2013 | Reproductive Health Outcomes in Eating Disorders | 2257 | Finland | to assess how eating disorders are related to reproductive health outcomes in a representative patient population. | Inpatient (Adult, Women) | Cross-Sectional | Reproductive health outcomes |
| Lundgren, et al. | 2010 | The Relationship of Night Eating to Oral Health and Obesity in Community Dental Clinic Patients | 174 | USA | to examine the relationship between night eating and oral health and obesity. | College (Adult, All Genders, Attending an academic faculty dental practice) | Cross-Sectional | Night eating behaviour, tobacco use, medical conditions, height, weight, oral health data |
| Lundgren, et al. | 2010 | Nocturnal Eating Predicts Tooth Loss Among Adults: Results from the Danish MONICA Study | 2217 | USA | to determine if nocturnal eating is related to tooth loss in a large, epidemiologic sample. | Community (Adult, All Genders) | Longitudinal (>5yr) | Oral health, eating behaviours |
| Maimoun, et al. | 2019 | Oral contraceptives partially protect from bone loss in young women with anorexia nervosa | 305 | France | to evaluate the potentially protective effects of oral contraceptives (OC) on bone loss in a large population of young women with anorexia nervosa (AN). | Inpatient (Young Adult, Women, Dx with AN) | Cross-Sectional | Areal bone mineral density (aBMD) |
| Martini, et al. | 2016 | Associations between eating disorder diagnoses, behaviours, and menstrual dysfunction in a clinical sample | 320 | Global | to investigate the following: the association between MD (i.e., primary and secondary amenorrhea) and (a) AN-R and AN-BP and BN and (b) ED behaviours in a clinical sample. | Outpatient (Adult, Women) | Cross-Sectional | ED symptoms, duration and frequency, menstrual function, |
| Mathisen, et al. | 2018 | Managing Risk of Non-Communicable Diseases in Women with Bulimia Nervosa or Binge Eating Disorders: A Randomized Trial with 12 Months Follow-Up | 164 | Norway | to compare CBT with an intervention combining physical exercise and dietary therapy (PED-t) and hypothesized that the PED-t would do better than CBT in lowering the risk of NCD both initially and longitudinally. | Outpatient (Adult, Women, Dx with BN or BED) | RCT | Body composition (BC) was measured by dual-energy X-ray absorptiometry. Measures of physical fitness (VO_2_peak and one repetition maximum (1RM) in squats, bench press, and seated row) were also recorded |
| Miller, et al. | 2011 | Effects of Risedronate and Low-Dose Transdermal Testosterone on Bone Mineral Density in Women with Anorexia Nervosa: A Randomized, Placebo-Controlled Study | 77 | USA | to determine whether antiresorptive therapy with a bisphosphonate alone or in combination with low-dose transdermal testosterone replacement would increase bone mineral density (BMD) in women with anorexia nervosa. | Inpatient (Adult, Women, Dx with AN) | RCT | BMD at the spine (primary endpoint), hip, and radius and body com-position were measured by dual-energy x-ray absorptiometry |
| Mumford, et al. | 2019 | Long-term Outcomes of Adolescent Anorexia Nervosa on Bone | 41 | Australia | To evaluate the natural history of bone health in patients with a previous diagnosis of adolescent-onset AN at 5 or 10 years post-diagnosis. | Inpatient (Young People, All Genders, Dx with AN) | Repeated Measure (with follow-up) | absorptiometry measurements of the total body, lumbar spine, and proximal femur, peripheral quantitative computed tomography at the radius and tibia, anthropometric measurements, serum biochemistry, fracture history, and a patient questionnaire |
| Nehring, et al. | 2014 | Long-term effects of enteral feeding on growth and mental health in adolescents with anorexia nervosa—results of a retrospective German cohort study | 208 | Germany | to find out whether EF improves growth and AN recovery and prevents psychiatric comorbidities. | Inpatient (Young People, Women, Dx with AN) | Longitudinal (>5yr) | Optimal growth, persistence of AN and occurrence of other psychiatric comorbidities |
| Nicolau, et al. | 2015 | Eating disorders are frequent among type 2 diabetic patients and are associated with worse metabolic and psychological outcomes: results from a cross-sectional study in primary and secondary care settings | 320 | Spain | to investigate the frequency of positive screening for ED, specifically binge eating disorder (BED), in a T2DM sample and analyse whether there are any differences among T2DM subjects with a positive screening for ED or BED. | Outpatient (Adult, All Genders, Dx with T2 Diabetes) | Cross-Sectional | Presence of an ED, screening of BED, sociodemographic, clinical and biochemical covariates |
| Nikniaz, et al | 2021 | A systematic review and meta-analysis of the prevalence and odds of eating disorders in patients with celiac disease and vice-versa. | 23 studies | N/A | to review studies that assessed the prevalence and risk of eating disorders (EDs) in patients with celiac disease (CD) and vice-versa. | | Systematic Review/ Meta-Analysis (combined) | Prevalence of EDS in patients with celiac disease |
| Nobles, et al. | 2016 | Association of Premenstrual Syndrome and Premenstrual Dysphoric Disorder with Bulimia Nervosa and Binge-Eating Disorder in a Nationally Representative Epidemiological Sample | 8694 | USA | to investigate the cross-sectional association between a lifetime history of PMS and PMDD, and a lifetime history of BN, BED and subthreshold BED in an ethnically diverse, nationally representative sample from the Collaborative Psychiatric Epidemiological Surveys. | Community (Adult, All Genders) | Cross-Sectional | PMS, PMDD and ED prevalence |
| O'Connor, et al. | 2016 | Refeeding Low Weight Hospitalized Adolescents with Anorexia Nervosa | 18 | UK | to test the hypothesis that refeeding adolescents with AN with a higher energy intake than what many guidelines recommend improved anthropometric outcomes without adversely affecting cardiac and biochemical markers associated with refeeding. | Inpatient (Young People, All Genders, Dx with AN) | RCT | Weight gain, QTc interval |
| Oudman, et al. | 2018 | Preventing Wernicke's encephalopathy in anorexia nervosa: A systematic review | 12 studies | N/A | to review the clinical characteristics of WE in AN, in order to raise the clinician’s index of suspicion about this neuropsychiatric diagnosis | Inpatient (Adult, All Genders, Dx with AN) | Review (Systematic) | Signs and symptoms of Wernicke's encephalopathy, sociodemographics, weight loss. |
| Pallier, et al. | 2019 | Dental and periodontal health in adults with eating disorders: A case-control study | 106 | France | to evaluate dental and periodontal health in anorexia nervosa and bulimia nervosa patients. | Inpatient (Adult, Women) | Case-control study | Dental attendance, oral hygiene behaviours, |
| Panico, et al. | 2018 | Oral Mucosal Lesions in Anorexia Nervosa, Bulimia Nervosa and EDNOS | 65 | Argentina | to describe oral lesions in patients with eating disorders (ED), including Anorexia Nervosa (AN), Bulimia Nervosa (BN) and eating disorders not otherwise specified (EDNOS). | Outpatient (Adult, All Genders) | Case-control study | Oral health, ED Dx and ED symptoms |
| Pasternak, et al. | 2012 | Obstetric and Perinatal Outcomes in Women with Eating Disorders | 117,875 | Israel | to investigate whether women with a history of eating disorders have an increased risk for adverse obstetric and perinatal outcomes. | Tertiary medical centre, pregnant women | Cross-Sectional | Pregnancy and birth complications |
| Peebles & Seike | 2019 | Medical Complications of Eating Disorders in Youth | N/A | N/A | outlines the medical assessment and treatment of malnutrition due to disordered eating. | | Review (Narrative) | Medical complications |
| Raevouri, et al. | 2014 | Highly Increased Risk of Type 2 Diabetes in patients with Binge Eating Disorder and Bulimia Nervosa | 2342 | Finland | to examine the prevalence and incidence of type 2 diabetes (T2D) in a large patient cohort treated for binge eating disorder (BED), bulimia nervosa (BN), and anorexia nervosa. | Inpatient & Outpatient (Young People - Adult, All Genders, Dx with AN, BN or BED.) | Repeated Measure (with follow-up) | Rates of type 2 diabetes before, during and after treatment, clinical covariates |
| Raj, et al. | 2012 | Hypomagnesemia in Adolescents with Eating Disorders Hospitalized for Medical Instability | 86 | USA | to determine the prevalence of hypomagnesemia (Mg ≤ 1.7 mg/dL) and clinical characteristics of adolescents hospitalized with a DSM-IV-diagnosed eating disorder who developed hypomagnesemia | Inpatient (Young People, All Genders, Dx with an ED) | Cross-Sectional | Clinical characteristics of patients with hypomagnesemia |
| Ridout, et al. | 2016 | Daily Laboratory Monitoring is of Poor Health Care Value in Adolescents Acutely Hospitalized for Eating Disorders | 196 | USA | investigates how the clinical practice guideline-recommended laboratory monitoring for refeeding syndrome impacts management and outcomes of adolescents with eating disorders hospitalized for acute medical stabilization and examines the value of laboratory monitoring (defined as the patient health outcomes achieved per dollar spent). | Inpatient (Young People, All Genders) | Cross-Sectional | Refeeding syndrome, laboratory costs |
| Rizzo, et al. | 2018 | Enteral Nutrition via Nasogastric Tube for Refeeding Patients With Anorexia Nervosa: A Systematic Review | 10 | N/A | to review the literature describing the efficacy, safety, tolerance, and long-term effects of nasogastric (NG) refeeding for patients with AN. | Inpatient (Adult, All Genders, Dx with AN) | Review (Systematic) | Efficacy, safety, tolerance, and long-term effects of nasogastric (NG) refeeding |
| Robinson, et al. | 2016 | A systematic review and meta-analysis of the association between eating disorders and bone density | 27 studies | N/A | to investigate bone mineral density (BMD) in individuals with anorexia nervosa (AN) and bulimia nervosa (BN) in comparison to healthy controls (HCs). | Dx with AN or BN | Systematic Review/ Meta-Analysis (combined) | Spine, whole body, hip and femoral neck bone mineral density |
| Robinson, et al. | 2017 | Pharmacological treatment options for low Bone Mineral Density and secondary osteoporosis in Anorexia Nervosa: A systematic review of the literature | 19 studies | N/A | to systematically review pharmacological bone active agents that increase BMD in women with AN | Outpatient (Adult, Women, Dx with AN, seeking tx for BMD) | Review (Systematic) | Data on BMD, fractures, biological markers (bone formation, metabolism, regeneration) |
| Rocks, et al. | 2014 | Nutrition Therapy during Initiation of Refeeding in Underweight Children and Adolescent Inpatients with Anorexia Nervosa: A Systematic Review of the Evidence | 7 studies | N/A | to systematically review, assess, and summarize the available evidence on the effect of differing nutrition therapies prescribed during refeeding on weight restoration in hospitalized children and adolescents (aged 19 years and younger) with diagnosed AN. | Inpatient (Young People, All Genders, Dx with AN) | Review (Systematic) | Nutrition treatment provided, weight, adverse events, length of treatment, |
| Rosen, et al. | 2016 | Liver Dysfunction in Patients with Severe Anorexia Nervosa | 181 | USA | to further elucidate the incidence and natural course of liver enzyme abnormalities in our patient population with severe AN that are undergoing the refeeding process. Furthermore, this article aims to identify potential risk factors associated with the development of elevated liver enzymes and to identify other medical complications that may occur in concert as a result of the liver dysfunction | Inpatient (Adult, All Genders, Dx with AN) | Cross-Sectional | Elevated aspartate aminotransferase and alanine aminotransferase, clinical covariates |
| Ruusunen, et al. | 2019 | The gut microbiome in anorexia nervosa: relevance for nutritional rehabilitation | N/A | N/A | to summarise the existing literature on the gut microbiome in AN and to consider the potential effects of nutritional rehabilitation on the gut microbiome in this context | | Review (Narrative) | Gut microbiome in AN and nutritional rehabilitation |
| Schalla & Stengel | 2019 | Gastrointestinal alterations in anorexia nervosa — A systematic review | 107 studies | N/A | to illustrate this complex interaction between the pathophysiology of AN and the GI tract. | Focused on AN and GI complains | Review (Systematic) | GI complaints in AN, Oral alterations in AN, GI motility in AN, Gastric motility, GI secretion in AN |
| Seidel, et al. | 2021 | A Systematic Review and Meta-Analysis Finds Increased Blood Levels of All Forms of Ghrelin in Both Restricting and Binge-Eating/Purging Subtypes of Anorexia Nervosa | 49 studies | N/A | to systematically review the literature to investigate the quality of evidence and the levels of the various forms of ghrelin present in plasma, in acute AN as well as during, and aftertreatment, compared to healthy controls (HC). | | Systematic Review/ Meta-Analysis (combined) | Change in ghrelin in AN |
| Sim, et al. | 2010 | Effect on bone health of estrogen preparations in premenopausal women with anorexia nervosa: a systematic review and meta-analyses | 6 studies | N/A | to estimate the influence of estrogen preparations (EP) on bone mineral density in women with AN | Dx with AN | Systematic Review/ Meta-Analysis (combined) | occurrence of bone fractures or measured bone mineral density at the radius, total hip, femur neck, or lumbar spine |
| Smith & Moran | 2021 | Gastrointestinal peptides in eating-related disorders | N/A | N/A | to explore the peripherally secreted gastrointestinal peptides (i.e., ghrelin, cholecystokinin, glucagon-like peptide 1, and peptide tyrosine tyrosine) that contribute to the control of appetite and discuss how these peptides or the signals arising from their release are disrupted in eating-related disorders across the weight spectrum, namely anorexia nervosa, bulimia nervosa, and obesity, and whether they are normalized following weight restoration or weight loss treatment. | Community (Adult, All Genders) | Review (Narrative) | N/A |
| Udo, et al. | 2014 | Menopause and Metabolic Syndrome in Obese Individuals with Binge Eating Disorder | 152 | USA | to compare the risk for metabolic abnormalities and MetS among postmenopausal women, premenopausal women, and age-matched men who were obese and sought treatment for BED | Outpatient (Adult, All Genders) | Cross-Sectional | ED Symptoms, Metabolic measures |
| Vajapeyam, et al. | 2018 | Magnetic Resonance Imaging and Spectroscopy Evidence of Efficacy for Adrenal and Gonadal Hormone Replacement Therapy in Anorexia Nervosa | 76 |  | to test the effects of DHEA+ estrogen/progestin therapy in adolescent girls with AN on bone marrow in the distal femur using magnetic resonance imaging (MRI) and spectroscopy. | Inpatient (Young People, Women, Dx with AN) | RCT | The primary outcome measure was bone marrow fat by MRI and magnetic resonance spectroscopy (MRS). |
| Vignaud, et al. | 2010 | Refeeding syndrome influences outcome of anorexia nervosa patients in intensive care unit: an observational study | 68 | France | to evaluate the prevalence and associated morbidity and mortality of AN in French ICUs. | Inpatient (Adult, All Genders, Dx with AN) | Cross-Sectional | Demographics, caloric intake, complications and refeeding syndrome |
| Weigel, et al. | 2019 | Severity of Somatic Symptoms in Outpatients with Anorexia and Bulimia Nervosa | 153 | Germany | to investigate the severity of somatic symptoms in outpatients with anorexia and bulimia nervosa. | Outpatient (Adult, All Genders, Dx with AN and BN) | Cross-Sectional | Somatic symptoms severity, days of sick leave, symptoms of depression, symptoms of anxiety, quality of life |
| West, et al. | 2021 | Gastrointestinal symptoms following treatment for anorexia nervosa: A systematic literature review. | 13 studies | N/A | to identify which treatments are effective in improving GI symptoms in patients with AN. | | Review (Systematic) | GI symptoms following AN treatment |
| Whitelaw, et al. | 2010 | Does Aggressive Refeeding in Hospitalized Adolescents with Anorexia Nervosa Result in Increased Hypophosphatemia? | 29 | Australia | to determine the incidence of hypophosphatemia (HP) in 12-18-year-old inpatients in order to inform nutritional guidelines in this group. | Inpatient (Young People, All Genders, Dx with AN) | Cross-Sectional | Number of past admissions, nutritional intake, weight, height, body mass index, and weight change at 2 weeks. |
| Whitelaw, et al. | 2018 | Predictors of Complications in Anorexia Nervosa and Atypical Anorexia Nervosa: Degree of Underweight or Extent and Recency of Weight Loss? | 171 | Australia | to compare total weight loss and recent weight loss with admission weight as predictors of physical and psychological complications. | Inpatient (Young People, All Genders, Dx with AN or AAN) | Cross-Sectional | Hypophosphatemia, clinical, anthropometric, and psychometric markers during admission. |
| Zaina, et al. | 2017 | Prevalence of idiopathic scoliosis in anorexia nervosa patients: results from a cross-sectional study | 77 | Italy | to evaluate the prevalence of idiopathic scoliosis in patients with AN. | Outpatient (Adult, Women, Dx with AN) | Cross-Sectional | Demographics, n of px dx with scoliosis and prevalence rates, BMI |
